# Case presentation and management of Lyme disease patients: a 9-year retrospective analysis in France

**DOI:** 10.3389/fmed.2023.1296486

**Published:** 2024-01-17

**Authors:** Emeline Perthame, Loïc Chartier, Jean-Claude George, Marie Varloud, Elisabeth Ferquel, Valérie Choumet

**Affiliations:** ^1^Bioinformatics and Biostatistics Hub, Institut Pasteur, Université Paris Cité, Paris, France; ^2^LYSARC, Centre Hospitalier Lyon-Sud, Pierre-Bénite Cedex, France; ^3^General Practitioner, Souilly, France; ^4^Ceva Santé Animale, Libourne, France; ^5^Unité Environnement et Risques Infectieux, Institut Pasteur, Université Paris Cité, Paris, France

**Keywords:** Lyme disease, Lyme borreliosis, epidemiology, multiple correspondence analysis, prevalence, symptoms, France

## Abstract

**Introduction:**

Lyme borreliosis (LB) is the most common vector disease in temperate countries of the northern hemisphere. It is caused by *Borrelia burgdorferi* sensu lato complex.

**Methods:**

To study the case presentation of LB in France, we contacted about 700 physicians every year between 2003 and 2011. An anonymous questionnaire was established allowing the collection of 3,509 cases. The information collected was imported or directly entered into databases and allowed identifying variables that were validated in a multiple correspondence analysis (MCA).

**Results:**

Sixty percent of the cases were confirmed, 10% were probable, 13.5% doubtful, 10.2% asymptomatic seropositive and 6.3% were negative. The clinical manifestations reported were cutaneous (63%), neurological (26%), articular (7%), ocular (1.9%) and cardiac (1.3%). Almost all patients were treated. When focusing more particularly on confirmed cases, our studies confirm that children have a distinct clinical presentation from adults. There is a gender effect on clinical presentation, with females presenting more often with erythema migrans or acrodermatitis chronica atrophicans than males, while males present more often with neurological signs or arthritis than females.

**Discussion:**

This is the first time that a comprehensive study of suspected Lyme borreliosis cases has been conducted over several years in France. Although we were not able to follow the clinical course of patients after treatment, these results suggest the interest of refining the questionnaire and of following up a cohort of patients over a sufficiently long period to obtain more information on their fate according to different parameters.

## Introduction

Lyme borreliosis is the most common vector disease in temperate countries of the northern hemisphere. This disease is distributed in all temperate zones of the Northern hemisphere and is caused by bacteria of the *Borrelia burgdorferi* sensu lato complex, which comprises 22 different genospecies (1). In the US, Lyme disease is caused by the dominant species *B. burgdorferi*, sensu stricto although a novel genospecies, *Borrelia mayonii*, has recently been identified (2, 3). In Europe however, several species of the complex: *B. burgdorferi*, sensu stricto *B. garinii*, *B. bavariensis*, *B. afzelii* and *B. spielmanii* were found responsible for human Lyme borreliosis. Two other species: *Borrelia valaisiana* and *B. lusitaniae* have been isolated from patients but their pathogenicity seems low (4, 5). In the early stage, the disease manifests as a skin lesion around the tick bite: erythema migrans (EM) (6). If left untreated, the disease may progress to an early disseminated stage with neurological (facial paralysis, meningoradiculitis), joint (arthritis), skin (borrelial lymphocytoma), or more rarely cardiac (conduction disorders) or ocular manifestations (7, 8). The late disseminated stage is characterized by cutaneous symptoms: acrodermatis chronica atrophicans (ACA), chronic arthritis and encephalopathy (9). The incidence of the disease in Western Europe is estimated to be approximately 22.05 cases per 100,000 person-years, but is certainly underestimated due to the lack of mandatory reporting of the disease in many European countries (10).

In France, Lyme borreliosis is mainly transmitted by the tick *Ixodes ricinus* found throughout the country except the Mediterranean coast and high altitude regions (11, 12). The animal reservoir, mainly sylvatic species, is vast and is made up of micromammals (rodents), birds and reptiles. Macromammals (deer and swine) are more involved in maintaining tick populations than as being a reservoir for *Borrelia* itself (13).

Lyme borreliosis currently represents a real public health problem and a potentially emerging disease mostly due to anthropogenic changes in the ecosystem and to a change in human behavior – leisure, afforestation etc. Global warming could allow a longer active period each year for the ticks leading to an acceleration of the life cycle, as well as an expansion of the latitude and altitude at which the ticks are found.

In France, the national surveillance is based on a network of sentinel general practitioners (14) and on clinicians or laboratories who willingly report the cases to the National Reference Center. In general practice, between 25,000 and 68,530 estimated cases of Lyme borreliosis have been diagnosed per year over the period 2009–2020. An increasing trend in the estimated annual number of cases has been observed since 2009 (15). Interestingly, the analysis of data on hospitalized LB cases does not show an increasing trend over the period 2005–2016 (16).

The *Borrelia* National Reference Center (NRC) at the Pasteur Institute contributed to the epidemiological surveillance of this zoonosis for the duration of its exercise from 2003 to 2011 by doing a survey with the collaboration of French medical practitioners that we contacted. 3,509 cases were thereby included in our study. The aim of our study was to have an estimation of the number of human cases as well as an analysis of the clinical forms of the disease and how cases evolved according to the years and regions in which they were reported. The medical charts of all patients suspected to suffer from Lyme disease collected by general practitioners, medical specialists, and biologists between 2003 and 2011 have therefore been reviewed for epidemiological data, clinical presentation, serological testing and treatment. LB-suspected cases were thereby analyzed in particular by using a new approach in the field of Lyme borreliosis, the multiple correspondence analysis (MCA), which allows the analysis of large amounts of data from questionnaires. The results showed that several regions of France, Auvergne-Rhône-Alpes, Grand-Est, Bretagne, Normandie and Ile-de-France were identified as more at risk than others. Case presentations evolved according to the years and regions in which they were reported. Our study confirms a different presentation of the disease depending on the sex and age of the patients. The duration of administration of antibiotics was in accordance with the recommendations of the French National Authority for Health. In addition, our results suggest the interest of refining the questionnaire and of following up a cohort of patients over a sufficiently long period to obtain more information on their fate according to different parameters.

## Materials and methods

### Ethics statement

The research project has not been assessed by an ethics committee, as the study was set up in France between the 1st January 2002 and 31st December 2011 and involved health data. The French law requiring the evaluation of research projects dates from 2012, and does not cover this type of research, which is described as non-interventional.

In this context, the project was not evaluated by the Institut Pasteur’s IRB either, as the latter was created in 2009, i.e., after the start of the research project. However, the project was subject and authorized by the Commission Informatique et Libertés (CNIL) in application of articles 40–1 and following of the law “Informatique et Libertés” (CNIL n°901,261).

Information was given orally by the doctor, accompanied by an information leaflet explaining the aims of the study, the voluntary aspect of participation and the right of refusal, and the legal framework. Participation was voluntary, and consent was obtained orally from adults or their parents if the patient was a minor. There was no legal requirement for written consent for this type of research, but it was noted by the investigator. Participants were able to ask questions and object to participation. Not participating in the study had no impact on patient management.

### Data source and surveillance

For this survey, the NRC has contacted about 700 physicians every year between 2003 and 2011 (medical specialist or general practitioners and biologists). An anonymous questionnaire was established. The information collected was imported or directly entered either by the NRC or by the practitioners into the EpiInfo (2003–2006) then Voozanoo (2007–2011) databases designed to collect data according to the NRC specifications.

3,509 case reporting files were collected. From these records, 2,104 cases of Lyme borreliosis were confirmed.

The inclusion of cases in our surveillance was based on definitions that have evolved over the years. Until the end of 2006, the definitions adopted were those of the European Union Concerted Action on Lyme Borreliosis (EUCALB) (17). They were then modified in 2007, according to the national consensus conference (18, 19). The definition of the cases is the following:

- Confirmed case: an EM of 5 cm or more in diameter (with or without serology), or arthritis, or secondary skin involvement, or cardiac involvement with positive serology, or neurological involvement with positive blood serology (ELISA and Western Blot) and positive CSF serology and lymphocytosis and intrathecal synthesis (ITS);
- Probable case: EM of 3–5 cm in diameter or without reported size with a notified history of tick bite or a notion of acarological risk (professional or leisure activity) or by secondary skin involvement with incomplete serology (WB data lacking), or by arthritis with positive serology without Western blot confirmation, or by neurological involvement with positive serology (blood or CSF), but in the absence of lymphocytosis or ITS;
- Doubtful/suspected case: EM of size less than 3 cm or without reported size with no notified history of tick bite or in absence of any notion of acarological risk (professional or leisure activity), or by secondary skin involvement with a doubtful serology, by neurological symptoms with no or partial serology results (blood or CSF), by rheumatological symptoms of arthritis in the absence of serology or not confirmed by Western Blot;
- Asymptomatic seropositive case: positive serology confirmed or not by Western blot but with no specific symptom of Lyme borreliosis specified at the time of consultation;
- Negative case: Negative serology, or positive serology but due to a cross reaction (syphilis or auto-immune diseases).

#### Data collected

They consisted in the cases seen in consultation from 2003, in the different departments where the doctors were willing to participate.

- Description of cases:

- Age, sex, date of diagnosis, notion of tick bite before episode (and if yes: date and place of the bite)
- Skin manifestations observed during the consultation and if yes: which: 1) EM (if yes: size, presence of a clear center, gradually expanding, single or multiple lesion, localization). Before 2007 the size requested was only greater than or equal to 5 cm, 2) borrelial lymphocytoma (BL), 3) ACA.
- Articular manifestations observed during the consultation and if yes which: existence of an acute arthritis or a chronic arthritis (if yes, the affected joints (knee, hip, elbow, other), if it was a mono-arthritis, an oligo-arthritis or a polyarthritis), or an arthralgia.
- Neurological manifestations observed during the consultation and if yes: existence of EM in the 2 months preceding the appearance of the manifestations (if yes, size), type of manifestation (facial paralysis, meningoradiculitis, clinical signs of meningitis, meningoencephalitis, radiculoneuropathy (and if yes: localization), other cranial nerve paralysis), lumbar puncture performed (if yes: date, number of cells per cubic millimeter, polynuclear and lymphocytes percentage), search for antibodies in the CSF (performed techniques: Elisa, Western blot, intrathecal antibody synthesis (ITS), and results) before 2007 the intrathecal synthesis was not required if the CSF was positive as well as the serology confirmed by Western blot: we counted them as positive. It is accepted, based on literature data, that lumbar puncture is not essential: 1) for meningoradiculitis or unilateral facial paralysis; 2) if there is a mention of EM within a compatible timeframe (maximum delay of 2 months between the onset of neurological symptomatology and the end of EM symptoms); 3) in the presence of positive serology with confirmation by Western Blot.
- Other manifestations described: cardiac manifestations (atrio-ventricular conduction disturbances, rhythm disturbances, myocarditis or pericarditis) or ocular manifestations (conjunctivitis, uveitis, keratitis).

#### Serology

In order to simplify, we chose to group results relative to serology in 10 classes according to the following definitions: class 1: serology not done; class 2: skin biopsy positive by PCR, or joint fluid positive by PCR; class 3: negative or doubtful serology; class 4: Positive serology (WB results not reported); class 5: positive serology, positive WB (IgM and/or IgG); class 6: positive serology, positive CSF, ITS not done + EM; class 7: positive serology, positive CSF, lymphocytosis or ITS made; class 8: positive serology, positive CSF, ITS not done; class 9: positive serology (cross reaction with syphilis or auto-immune disease); class 10: serology not specified.

#### Incidence

The incidence estimate is based on the census of the number of definite cases. The extrapolation to the entire population of the region is made from the rate of participating physicians. During 9 years, the *Borrelia* NRC at the Pasteur Institute studied the incidence of the disease and analyzed the frequency of clinical forms in three regions: Grand-Est, Auvergne-Rhône-Alpes, Normandie.

### Multiple correspondence analysis

MCA is an extension of the well-known PCA (Principal Components Analysis) specifically dedicated to the analysis of qualitative data such as responses to a questionnaire. This allows to simultaneously analyzing the patient profiles in both a comprehensive and a multivariate way. This method is implemented in FactoMineR R package (20). We selected 32 variables as active in the analysis used to compute the principal dimensions and 15 variables as supplementary, used for further investigation. MCA is a dimension reduction method, so it provides a low dimensional representation of a multidimensional dataset while preserving distances among variables (questions of the questionnaire) and individuals (patients). The patients exhibiting similar symptoms, exposition, treatment, serology are therefore close to each other. Interestingly, MCA provides a dual representation of both variables and patients so one can interpret similarities among patients as well as among variables. Methodological details about the method were described by Greenacre and Blasius (21) and interpretation keys by Florensa et al. (22). As an extension of PCA, MCA computes linear combinations, the so-called principal dimensions or principal components, of the variables of a dataset to maximize the distance (entropy) between individuals. The advantage of representing a multivariate dataset on dimensions computed by a factorial analysis such as MCA is to identify which variables are important in a dataset to discriminate clusters of patients. The interpretation of such analysis is mainly graphical, based on the so-called scatter plot of the patients projected onto principal dimensions. In addition, quantitative quality measures such as Eta2, v-tests and variables help to rank the variables under study, identify subgroups of patients and associations between variables. For each principal components, we denote by Eta2 a measure between 0 and 1 indicating how much a variable discriminates the observations (say, e.g., symptom.definition on Dimension 1); while contributions denote values between 0 and 100% indicating how much a level of a variable is associated to the aforementioned component (say, e.g., dermatological cases of variable symptom.definition). At last, v-tests are convenient for interpretation similarly to contributions as they can be assimilated to Z-scores (meaning that one can focus on v-tests higher to 2 in absolute value). For each figure representing the patients, missing values or unrelevant answers (e.g., when no bite location were recording because the patient was not bitten) are not shown on the graphs while accounted for in the analysis as a level of the corresponding variable. The percentages indicated in brackets on each MCA projection is the percentage of variance explained by the given principal component. All the results can be found in https://lymeborreliosis.shinyapps.io/ShinyApp/.

### Evaluation of the distribution of dates of bites and diagnostic

Kurtosis is a statistical measure that describes the shape of a probability distribution. It is a measure of the degree of peak or flatness of a distribution relative to the normal distribution. A high kurtosis value indicates a more peaked distribution, while a low kurtosis value indicates a flatter distribution. Here, we use it to study the distribution of the dates of tick bites and diagnostic.

## Results

### Epidemiological data of the 3509 cases

#### File collection and participating practitioners

Three thousand five hundred and nine reporting files have been collected during 9 years. The number of patient files has increased between 2003 (100) and 2006 (354) to reach a maximum in 2009 (621) and 2010 (634) (Supplementary Table S1). Note that the database software used to collect the files changed during the study. No effect of this modification was detected by the MCA (data not shown). The participating practitioners were mainly general practitioners or internists. 77% were working in ambulatory medicine and 23% in hospitals.

#### Distribution of the cases in France

The reported cases were distributed into 13 regions mainly in Auvergne-Rhône-Alpes (37.8%), Ile-de-France (24.2%), Grand-Est (19.5%) and Normandie (9.2%) (Table 1). The case distribution is shown by region and department on the Shiny-App.

**Table 1:** Number of cases reported by region.

Region	Cases number (%)
Auvergne-Rhône-Alpes	1,325 (37.8)
Bourgogne-Franche-Comté	6 (0.2)
Bretagne	229 (6.5)
Centre-Val-de-Loire	13 (0.4)
Corse	1 (0)
Grand-Est	683 (19.5)
Hauts-de-France	28 (0.8)
Ile-de-France	848 (24.2)
Normandie	322 (9.2)
Nouvelle-Aquitaine	24 (0.7)
Occitanie	7 (0.2)
Pays-de-la-Loire	12 (0.3)
Provence-Alpes-Côte-d’Azur	4 (0.1)

#### Description of the population

**Figure 1:**
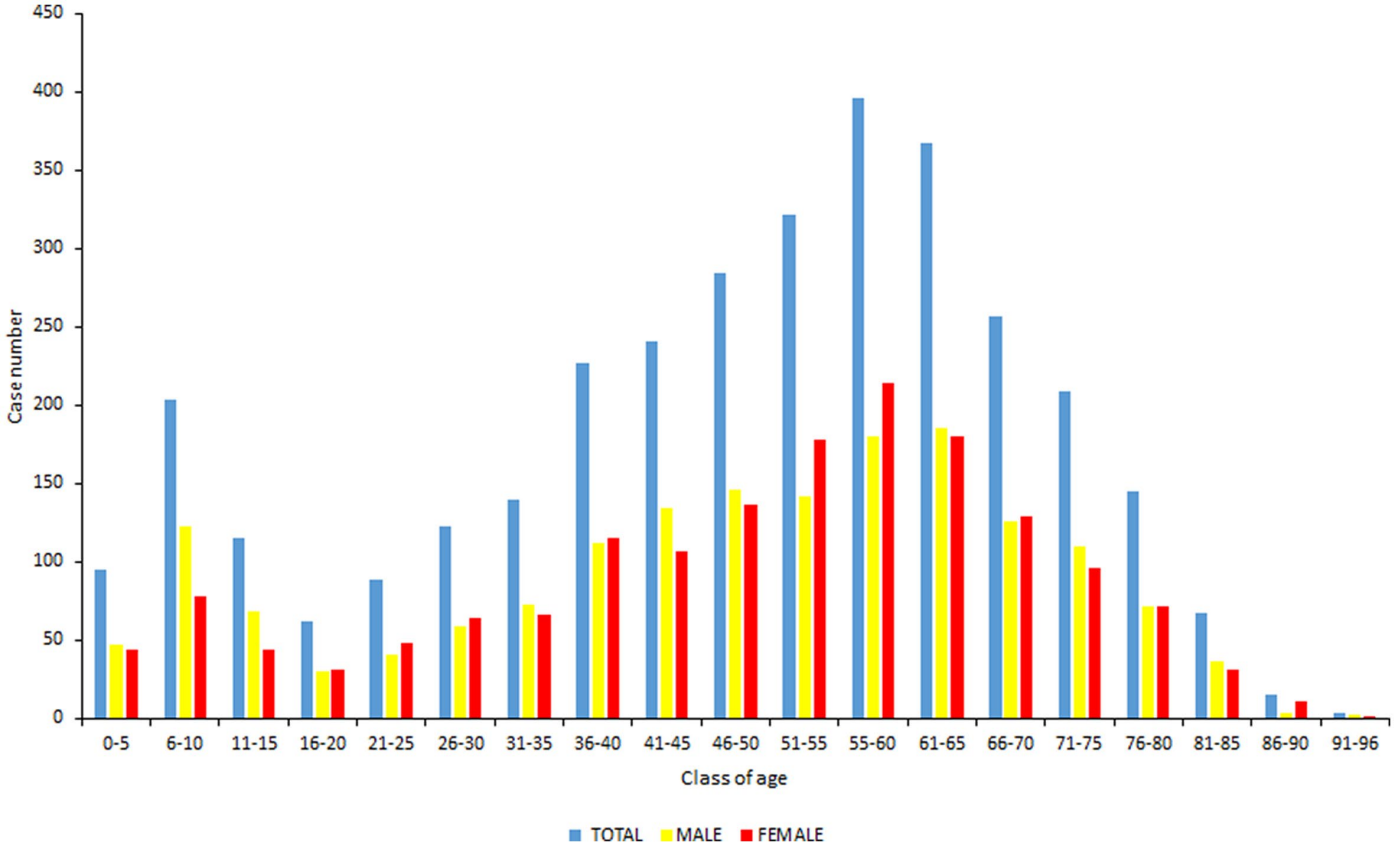
Repartition of the cases as function of classes of age. In blue: total population, in yellow: male population, in red: female population.

Among the 3,509 subjects, 50.2% were men and the average age was 48 years. The repartition of the cases was studied as function of classes of age. As shown in Figure 1, a bimodal repartition can be observed with two peaks among children of 6–10 years of age and adults 55–60 years of age. Dimension 2 of the MCA shows a moderate discriminant effect of patients age, specifically between <16 and > 16 years old patients (Eta2 = 0.24) (Supplementary Figure S1A) with young patients located in grey at the bottom of the two clusters of the scatter plot. We further analyzed the ratio female/male for each class of age.

For classes 5–10, 11–15, and 41–45, the number of males were statistically superior to females (*p* < 0.00001, *p* = 0.0013 and *p* = 0.014 respectively). It is the opposite for 51–55, 55–60, and 86–90 age groups for which the number of females was superior to males (*p* = 0.004, *p* = 0.015, and *p* = 0.01 respectively).

We then analyzed the occupations and hobbies of the study population that could increase exposure to ticks. Concerning exposure to occupational risks, we observed that 10.8% of the subjects were at risk, the main profession (63%) being farmer. Dimension 2 of the MCA shows a moderate discriminant effect of profession at risk (Eta2 = 0.15) (data not shown). The risk ratio for the profession at risk showed a significant difference in favor of men (11.6% versus 4% for women). There is a bias since mostly men practice this profession. The majority of the other patients practiced a hobby at risk of exposure to tick bite (96.8%), the main one being walking in the forest (69.6%) (Supplementary Table S2). There is no difference between men and women in this population.

#### Tick bite

69.4% of the subjects noticed a tick bite before the symptoms (Supplementary Figure S1B). When documented (1,184/1,891) (69.4%), it was located mainly in the lower limbs (56.3%), then on the trunk, abdomen (18.8%) or the upper limbs (12.8%) (*p* < 0.00001 lower limb > truck > upper > head > others). For children, we can see than most of the bites were detected on the head (Supplementary Figure S1C).

#### Seasonal distribution of tick bite and diagnostic date

**Figure 2:**
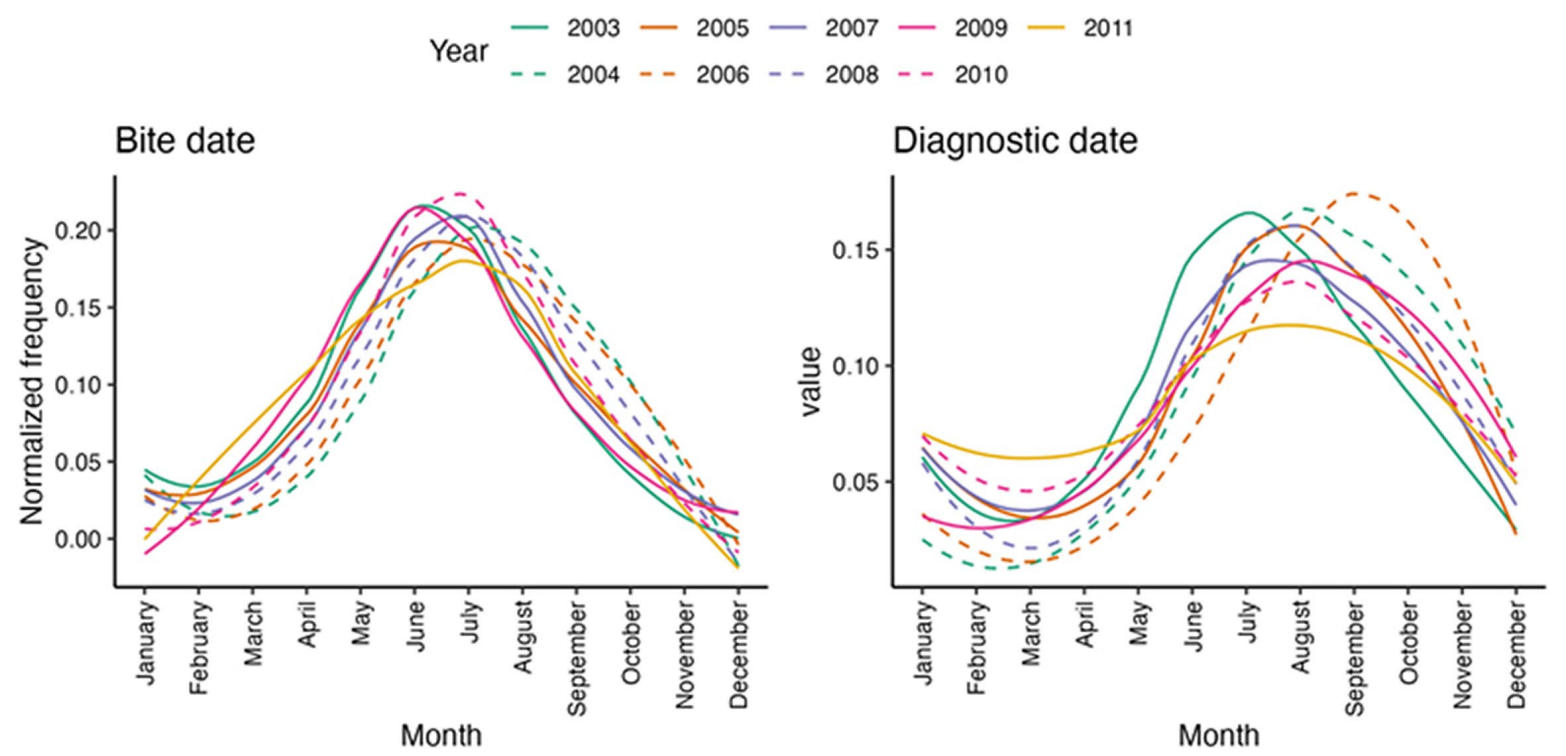
Distribution of dates of tick bite and of diagnosis as function of the year.

The seasonal distribution of Lyme borreliosis cases and tick bite is shown as function of the year in Figure 2. We can see that the bite and mostly diagnostic dates tend to be spread out over the whole year after 2008, whereas they are more concentrated in the summer before 2008, as confirmed by the Kurtosis coefficient that tends to decrease with years, especially for diagnostic dates (data not shown). The observation is less clear for bite dates.

#### Classification of the cases

Among the 3,509 reporting files and according to the classification of the cases described in the Material and Method section, a great majority were confirmed (60%) while 10% were probable, 13.5% were doubtful, 10.2% were asymptomatic seropositive cases and 6.4% were negative. The percentage of confirmed cases evolved as function of the year. In 2003, 2008, 2009, and 2010, we observed ratios ranging from 50 to 60% that were statistically inferior to what was observed in 2004, 2005, 2006, and 2007, where they varied between 70 to 80% (*p* < 0.00001). In good agreement, the percentage of probable, asymptomatic seropositive and doubtful cases significantly increased in 2008, 2009, 2010–2011, respectively.

**Figure 3:**
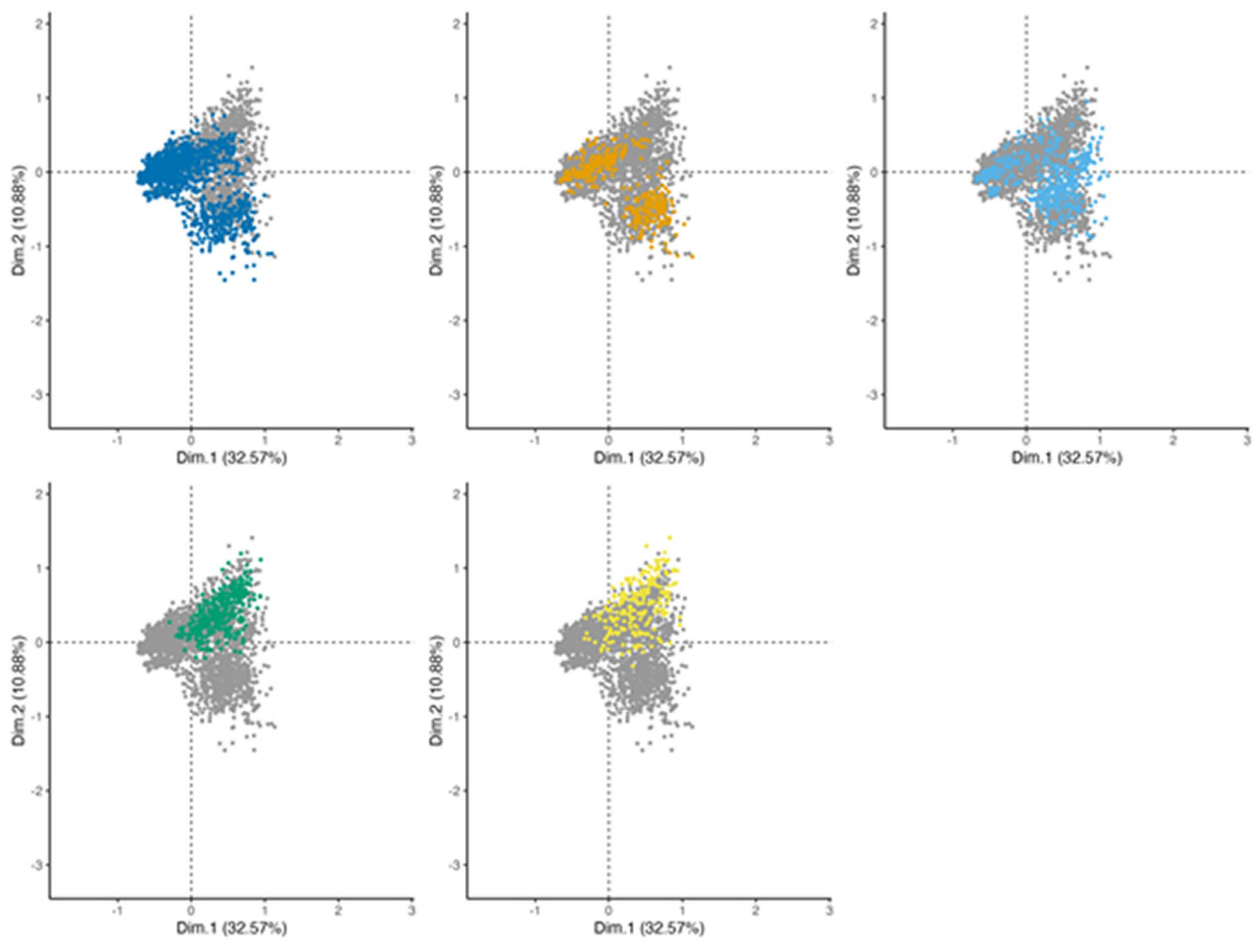
MCA projection of patients on dimensions 1 (*x*-axis) and 2 (*y*-axis), showing the case definition of the reported patients’ files. To ease the visualization, the projection is stratified with each panel representing one case definition level (dark blue: confirmed, orange: probable, light blue: doubtful, green: asymptomatic seropositive, and yellow: negative), other levels represented in grey for comparison.

We tested whether the MCA was able to robustly retrieve the case segregation from the clinical variables as we provided to MCA only clinical variables (active variables) and cases classification was used as supplementary meaning that it was not used for computation but only for visualization. The interest of this approach is to verify whether the classification established in this article based on clinical variables is relevant for identifying clusters of individuals that are quite distinct at the scale of the data set. Figure 3 shows that the case information was retrieved by the 2 first dimensions of the MCA, showing an interesting “gradient” of case distribution from the left (confirmed cases) to the upper right (asymptomatic seropositive and negative cases) through probable cases on the bottom right and the doubtful cases. Confirmed cases are split into two visible clusters: one cluster located on the left; a second cluster located on the bottom-right of the figure. The structure of these two clusters will be further described when the symptoms recorded will be analyzed.

Note that probable cases have coordinates close to confirmed cases, showing an overlap between these two clusters, which demonstrates the difficulty to segregate confirmed and probable case. The analysis shows little difference between asymptomatic seropositive and negative cases as the two clusters are completely overlapping. The observation is similar on the other dimensions computed by the MCA.

Doubtful cases are difficult to classify as the individuals locate in the entire scatter plot, confounded with other case definition, exhibiting the uncertainty around the infection status of these patients.

### Clinical data of the 3509 cases

#### Systemic symptoms

At the time of diagnosis, 32.7% of patients had systemic manifestations including mainly asthenia, myalgia, arthralgia and fever. Association of several systemic symptoms was also reported as shown in Table 2.

**Table 2:** Systemic symptoms reported in the collected patient file.

Systemic symptoms	1,142/3,496 (32.7%)
**Arthralgia**	**13%**
**Asthenia**	**16%**
Asthenia + arthralgia	4%
Asthenia + myalgia	8%
Asthenia + myalgia+arthralgia	3%
Fever (>37.7°C)	7%
Fever + arthralgia	1%
Fever + asthenia	3%
Fever + asthenia + arthralgia	1%
Fever + asthenia + myalgia	4%
Fever + asthenia + myalgia + arthralgia	2%
Fever + myalgia	2%
Fever + myalgia + arthralgia	0.4%
Headache	1%
**Myalgia**	**15%**
Myalgia + arthralgia	3%
Others	5%
Others + arthralgia	1%
Unspecified	7%
Unspecified + arthralgia	2%

We selected the cases for which this data was mentioned (3,496/3,509).

Interestingly, systemic symptoms are well represented by the MCA on the 3 first dimensions (cumulated Eta2 = 0.45). On dimensions 1 and 2, headache segregates in the lower right corner while most of other systemic symptoms tend to cluster in the upper right of the graph (Supplementary Figure S2).

If we look at the topology of the case definition on dimensions 1 and 2 (Figure 3), we can see that asymptomatic seropositive and negative patients (upper right) cluster with more systemic symptoms than confirmed and probable cases (Supplementary Figure S2). We checked the proportion of systemic symptoms in the different cases and proved that confirmed cases (25.3%) have the fewest systemic symptoms while asymptomatic seropositive cases have the most (66.1%) (*p* < 0.00001).

#### Lyme borreliosis symptoms

Among the 3,509 reporting cases, 62.6% of patients had a cutaneous manifestation, the most frequent being EM (89.4%). Twenty-six percent of the patients had neurological manifestations, 6.9% of the subjects had joint manifestations, 1.9% had ocular manifestations and 1.3% had cardiac manifestations.

**Figure 4:**
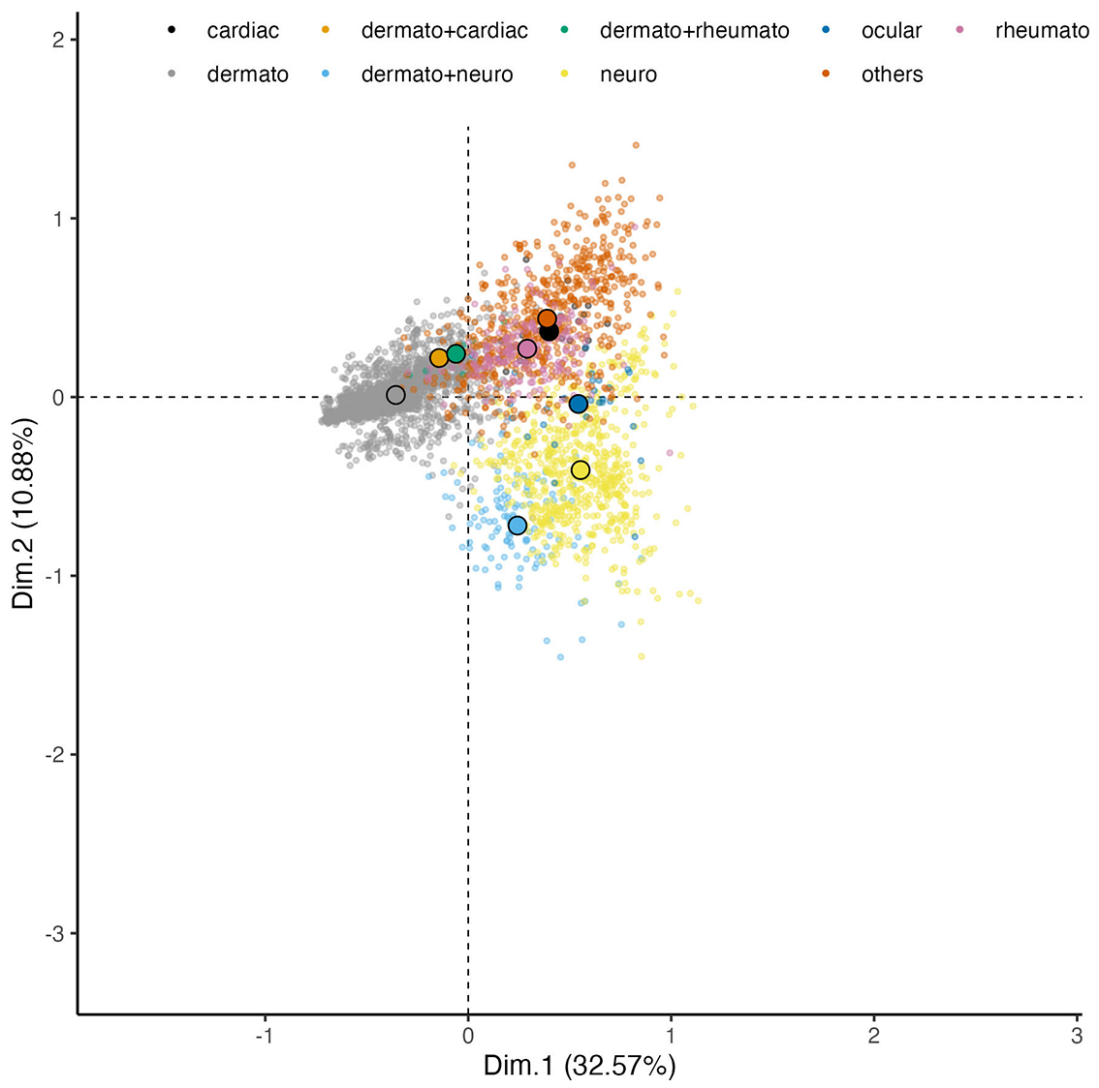
MCA projection of the Lyme-related symptoms on dimensions 1 (*x*-axis) and 2 (*y*-axis). Each small transparent dot represents the coordinates of the patients having this symptom. Dots are colored by symptoms. To ease the visualization due to the overlap, the large colored dots circled in black represent the gravity centers of each symptom (average of the coordinates of patients by symptoms).

We found that neurological (Eta2 = 0.39 and 0.35 for dimensions 1 and 2 respectively), cutaneous (Eta2 = 0.68 for dimension 1) as well as rheumatoid (Eta2 = 0.35 for dimension 3) symptoms are strong contributors to the analysis on the 3 third dimensions. As shown in Figure 4, dimensions n°1 and 2 of MCA showed a robust segregation of case definition in good agreement with the structure of cutaneous, neurological, and in a lesser extent of joint cases, which are better represented on dimension 3. Dermatological symptoms characterize a high part of confirmed cases. Note that patients with dermatological associated with cardiac symptoms or articular symptoms are clustered with patients with only dermatological symptoms.

Neurological symptoms characterize probable cases and a second part of confirmed cases with an association to dermatological symptoms (Figures 3, 4), which suggests that patients presenting both neurological and dermatological symptoms are closer to neurological cases than dermatological ones, probably due to the serological status of these cases. Cardiac, rheumatological and ocular cases are segregated on the right panel.

##### Cutaneous manifestations

The main cutaneous manifestation was EM (89.4%). Very few patients had BL (2%), acrodermatitis (2.6%) and only 1% cases of multiple erythema migrans (MEM). This is supported by the MCA where EM are located on the left of the figure, contributing strongly to the first dimension (contribution = 4.38%) while other cutaneous symptoms are rare and spread on the whole scatter plot, with low contributions (contributions <0.05%) for the first 2 dimensions (Supplementary Figure S3A). Women (54.4%) were most affected than men (45.6%) (*p* < 0.00001) (Supplementary Figure S3B). A large majority (77.3%) of the EM observed were not accompanied with general signs (*p* < 0.00001).

Other skin manifestations (morphea, dermatitis, alopecia, skin eruption, petechia, edema, eczema) were identified in asymptomatic seropositive and negative cases.

##### Neurological manifestations

**Figure 5:**
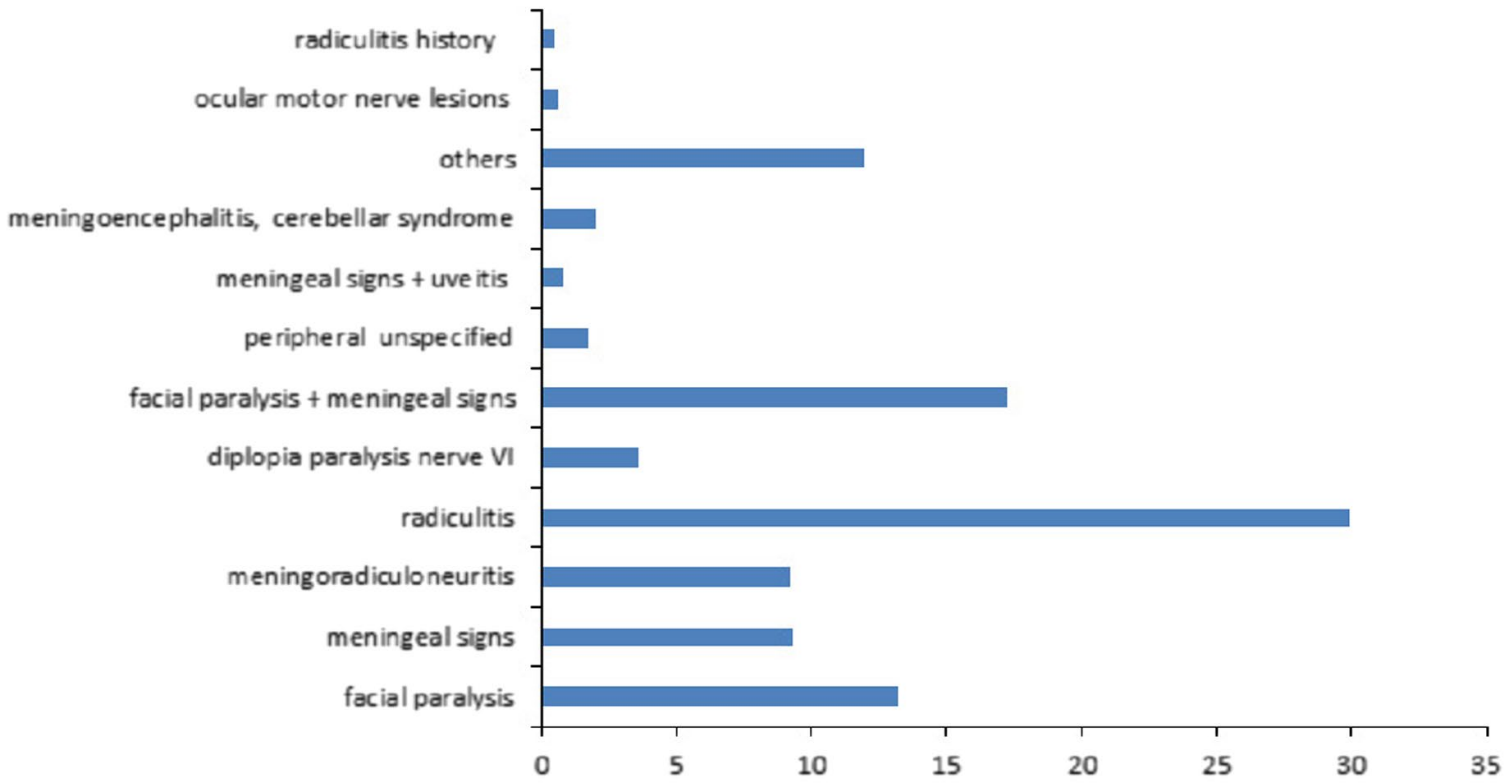
Description of the neurological manifestations observed in the 3,509 patient files.

**Figure 6:**
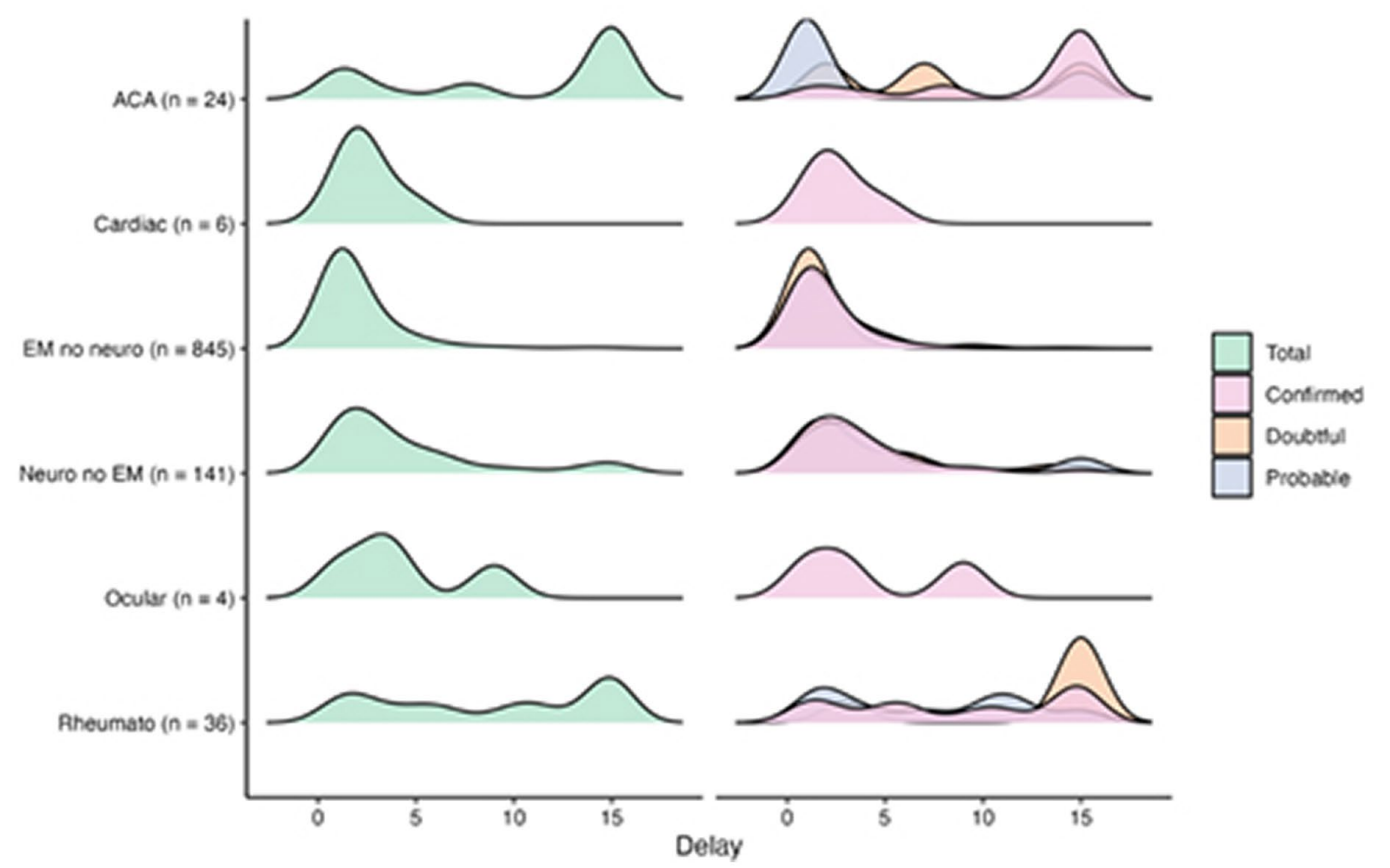
Delays distribution across case definition and symptoms.

Among the 3,509 patients, 915 patients (26%) had neurological manifestations. The main neurological manifestations were facial paralysis alone or with meningeal signs (34.9%) and radiculitis (30%) (Figure 5). Neurological symptoms are well represented by the MCA on the two first principal components with high Eta2 values (0.75 on dimensions 1 and 2) (Figure 6).

Among the subjects having had a facial paralysis whose lateralization was documented (11/121; 9%), more than half (63%) was located on the right side, 27% on the left.

##### Joint manifestations

We counted few patients (7.2%) with specific Lyme rheumatoid signs. Joint manifestations include 71.3% of acute arthritis. These patients presented 51.4% of monoarthritis among which 62% were localized in the knee, 21.8% presented oligoarthritis, 16.2% suffered from polyarthritis and 10.6% were not indicated. Among the patients having a chronic arthritis (25.1%), 41.3% were monoarthitis of which 65.4% reached the knee.

##### Cardiac and ocular manifestations

Few subjects had cardiac manifestations (1.3%). They consisted in 31.1% of atrioventricular block, 28.9% of cardiac muscle inflammation, and 26.7% of heart rhythm disorder. The ocular manifestations represented only (1.9%) of the cases, manifesting in majority by 38.2% uveitis, 38.2% others like vision disorder, 8.8% choroiditis and 14.7% unspecified.

##### Delays distribution across case definition and symptoms

We analyzed the chronology of appearance of the various symptoms (Figure 6). We were able to retrieve 1,268 delays between tick bite and diagnostic out of the 3,509 cases (36%) (71.7% in confirmed, 8.8% in probable, 9.3% in doubtful, 4.6% in asymptomatic seropositive and 5.7% in negative cases). Smoothed density curves show the distribution of delays between bite and diagnostic dates in number of months (delays higher than 15 months are aggregated) across type of cases and case definition. Dermatological, neurological, and cardiac symptoms occurred during the first 5 months after the bite, whether the cases were confirmed probable or doubtful. Several peaks were observed for ocular and rheumatological symptoms. Regarding ACA, the vast majority of confirmed cases manifested late while three peaks appeared between 0 and 5 months, between 5 and 10 months and between 10 and 15 months for doubtful cases.

### Treatment of the 3509 cases

Whatever the diagnosis was, almost all of the patients received antibiotic treatment (94.4%). Oral beta-lactam represented 51.5%, parenteral beta-lactam 22.3% followed by cyclin (18%). Macrolides represented only 0.9% of the treated patients. 7.1% of the patients received two treatments. The treatment status (yes/no) and treatment type are well represented by the MCA (Eta2 = 0.21 and 0.48 respectively]) on the two first dimensions (Supplementary Figure S4A). The analysis retrieves that neurological cases tend to be treated by parenteral antibiotics, with patients located on the bottom-right corner of the MCA, while oral route antibiotics are administered in EM cases, with patients located on the left of the MCA (Figure 6 and Supplementary Figure S4A). This observation is confirmed on raw data (Supplementary Figure S4B). We also observed a difference of treatment between adult and children (Supplementary Figure S4B). There is very little use of cyclin in children (4.2%) compared with adults (19.8%), because of the side effects in this age group (*p* < 0.0001). Parenteral administration of beta-lactam antibiotics is more commonly used in children (38.5%) than in adults (20.3%) (*p* < 0.0001).

### Serology of the 3509 cases

**Figure 7:**
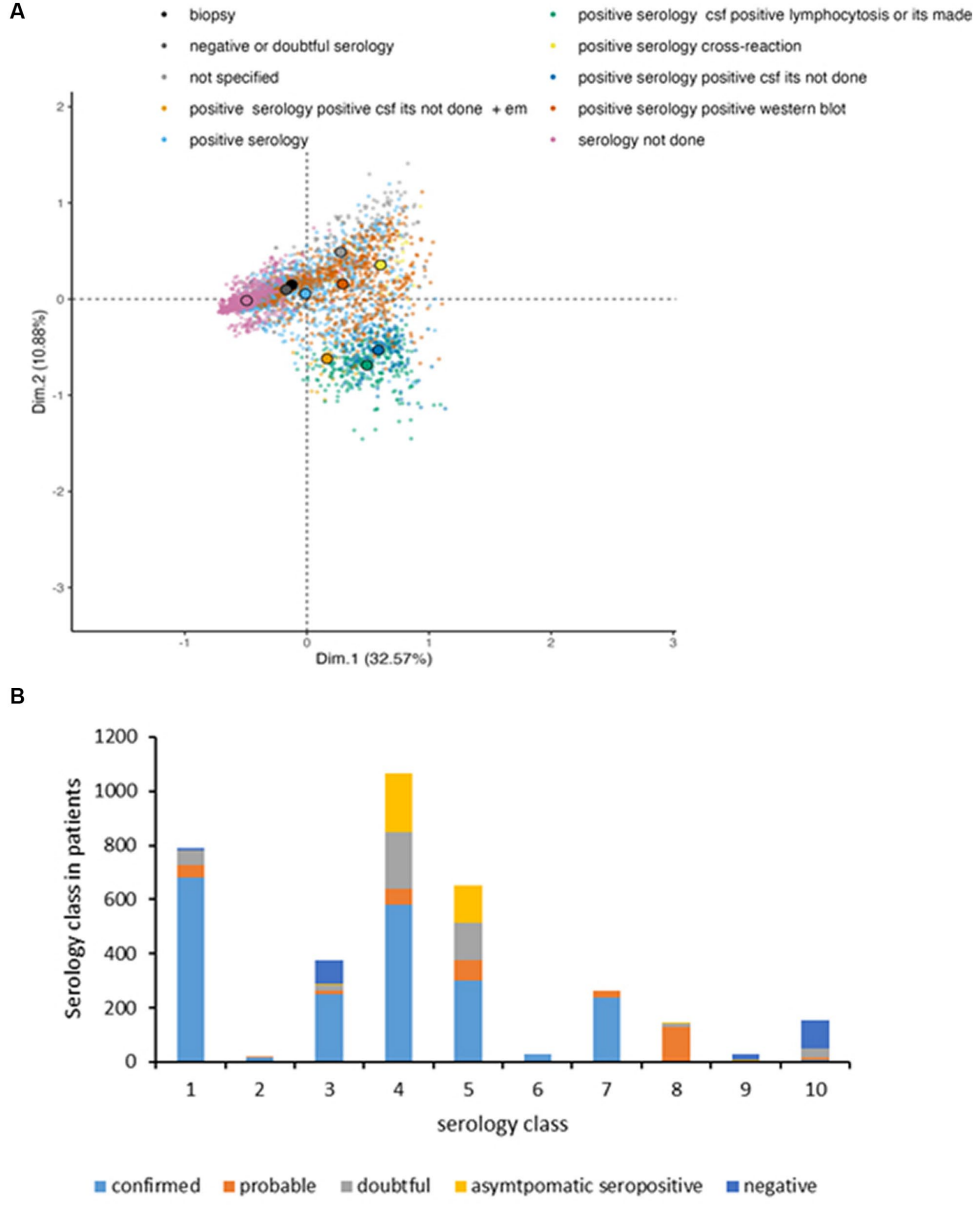
Serological tests performed as function of the symptoms. **(A)** MCA projection of serological diagnostic on dimensions 1 (*x*-axis) and 2 (*y*-axis). Each small transparent dot represents the coordinates of the patients having this serology performed. Dots are colored by symptoms. To ease the visualization due to the overlap of patients, the large colored dots circled in black represent the gravity centers of each serological test (average of the coordinates of patients by serological test). **(B)** Type of serology as function of case definition: (1) serology not done, (2) biopsy, (3) negative or doubtful serology, (4) positive serology, (5) positive serology confirmed by WB, (6) positive serology and CSF, ITS not done +EM, (7) positive serology, positive CSF, lymphocytosis or ITS made, (8) positive serology positive CSF, ITS not done, (9) positive serology due to nonspecific cross reaction, and (10) not specified.

We then examined the results of serology for the 3,509 cases. Unsurprisingly, the serology type and the symptom definition appear to correlate with case definition on dimensions 1 to 3 (see Figures 7A, 3) and Eta2 [0.55, 0.52, 0.22]) as the case definition is partially based on serology type.

We focused on the type of serology performed according to the classification of the cases and the symptoms recorded (Figure 7B). In the first case, among the 22.5% of untested serology, 86.3% were confirmed cases presenting EM (100%), 5.6% were probable cases with 100% EM, 7.1% of doubtful cases with 80.4% EM and the negative cases represent 1% (12.5% EM).

The second class regrouped 0.5% of the cases and consisted in 20% joint fluids (1 chronic and 2 acute arthritis) and 80% skin biopsy. Interestingly, *Borrelia afzelii* was identified in one case of acute arthritis and *Borrelia valaisiana* and *Borrelia afzelii* were detected in cases of ACA.

In the third class (10.7% of the cases), EM was the major presentation in confirmed, probable and doubtful cases. Class 4 (30.3%) consisted in 27.6% of confirmed cases (89.2% EM), 16.3% of probable cases, 44.5% doubtful, 60.1% of asymptomatic seropositive cases. Class 5 (18.5%) contains confirmed cases (14.3%), in which various case presentations were observed (33.2% acute arthritis, 16.6% EM, 10% ACA, 9.6% chronic arthritis, 5% cardiac symptoms and 5.6% ocular signs). In the probable cases, 73.6% of EM were identified. The doubtful cases presented mostly neurological symptoms (96%). Class 6 (0.8%) is mainly represented by confirmed cases that consisted in 100% of neuroborreliosis. Class 7 (7.5%) included 100% confirmed neuroborreliosis cases among which were 33.6% of EM. Class 8 (4%) contained 100% of probable neuroborreliosis. Class 9 (0.8%) consists in 67.9% negative cases and 21.4% of asymptomatic seropositive cases. Class 10 (4.4%) included 69.2% of negative and 5.1% of confirmed cases (100% EM).

### Confirmed cases

We then analyzed more particularly the confirmed cases that represent 60% of the reported cases. We evaluated the incidence of the confirmed cases in departments of Auvergne-Rhône-Alpes, Grand-Est and Normandie. In the department of Meuse (monitored for 9 years), the estimated incidence remained high (194/100,000 inhabitants in 2010) (data not shown). The estimated average incidence in Auvergne-Rhône-Alpes decreased significantly in 2010 compared to 2009 to return to the figures obtained in 2008 (78/100,000 vs. 92/100,000 inhabitants) with an increase in Puy-de-Dôme, a decrease in Cantal, and a stable number of cases in Allier. In three departments of Normandie (Calvados, Orne, Manche), the incidence was moderate (10 per 100,000 inhabitants). It was higher in the other two (Eure, Seine-Maritime) (30 per 100,000 inhabitants), particularly in Seine-Maritime compared to the other departments. The incidence clearly decreased in 2009 compared to 2008. This decrease was especially marked in Eure, as we already noted in 2009. The age of the study population was 49 ± 20.7 [1–95]. Women were more numerous than men (*p* = 0.01). We then analyzed the repartition of the confirmed cases as function of class of age. The number of diagnosed cases shows that there is a significant difference (*p* < 0.00001) showing that most cases were reported in classes 41–50, 51–60 450 and 61–70.

We also analyzed occupations and hobbies of the study population. Concerning exposure to occupational risks, we observed that 9.7% of the subjects were at risk, the main profession (68.2%) being farmer. The other patients practiced a hobby at risk of exposure to tick bite (98.9%), the main one being walking in the forest (68.7%). Interestingly, 73.2% of the confirmed cases reported a tick bite before the diagnostic of Lyme borreliosis.

#### Clinical presentation

Twenty-five percent of the confirmed cases presented nonspecific systemic symptoms, asthenia being the most prevalent compared to the other manifestations. Interestingly, we observed that fever and headache were statistically frequent in confirmed cases (*p* = 0.0002 and *p* < 0.00001 respectively) and associated with neurological symptoms. 80% of the confirmed cases presented skin manifestations (EM, MEM, ACA, BL), 12% had neurological manifestations and 6.6% suffered from Lyme arthritis. The other signs were observed in less than 5% of the reported cases. Delayed neurological manifestations were only reported in 0.2% of the cases.

**Figure 8:**
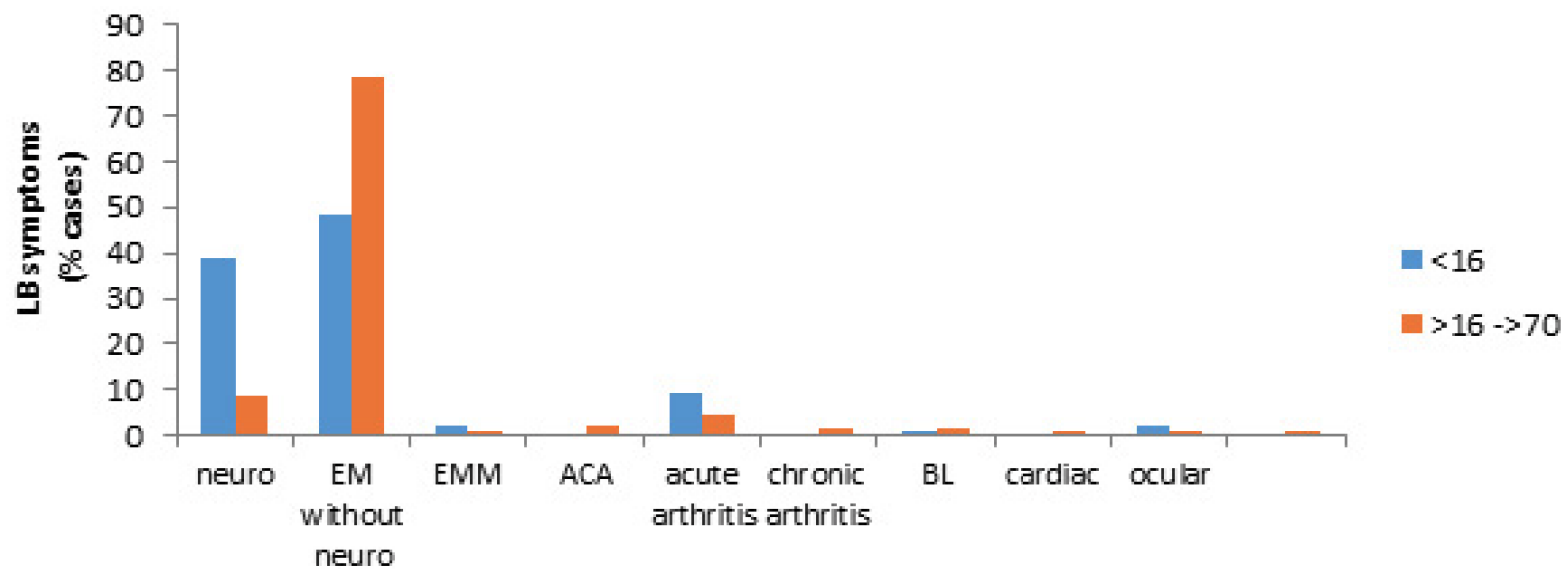
Clinical presentation in children (<16 years) and in adults (>16 years). Children are presented by blue bars and adults by orange bars.

We then compared the case presentation between children and adults. As shown in Figure 8, neuroborreliosis were more frequent in children than in adults (*p* < 0.00001) whereas this is the opposite when skin manifestations were concerned (*p* < 0.00001). For the class of children <16 years old, neurological signs (39%) and EM (48.6%) were higher than acute arthritis cases (9.5%) (*p* < 0.00001) whereas for adults, EM cases (78.9%) are statistically superior to the other signs (*p* < 0.00001). They were followed by neurological cases (8.8%) and rheumatoid cases (6.1%) that were superior to the other signs (*p* < 0.00001). ACA, chronic arthritis and cardiac signs were observed only in adults. Ocular signs always accompanied neurological signs in children.

##### Non disseminated phase symptoms: EM

We first analyzed whether a difference of prevalence could be observed over the years of study. Interestingly, a high prevalence of EM was observed in the years 2004 to 2007 whereas a low prevalence was observed in 2011 (data not shown). We then investigated the characteristics of the EM. We observed that EM was more frequently observed in female (55.1%) than in male (44.9%) (*p* < 0.00001). Only 15.5% of the EM were accompanied by general symptoms, the principal signs being asthenia, myalgia, fever and arthralgia. 5.6% of EM were associated with confirmed neuroborreliosis and 0.2% with confirmed acute arthritis. We then verify whether the age could interfere with the repartition of the EM case number. Indeed, a significant difference appears (*p* < 0.00001), the age classes 51–60, 61–70 were those for which the highest number of EM cases were observed. The class <16 years and 16–30 years were significantly lower.

**Figure 9:**
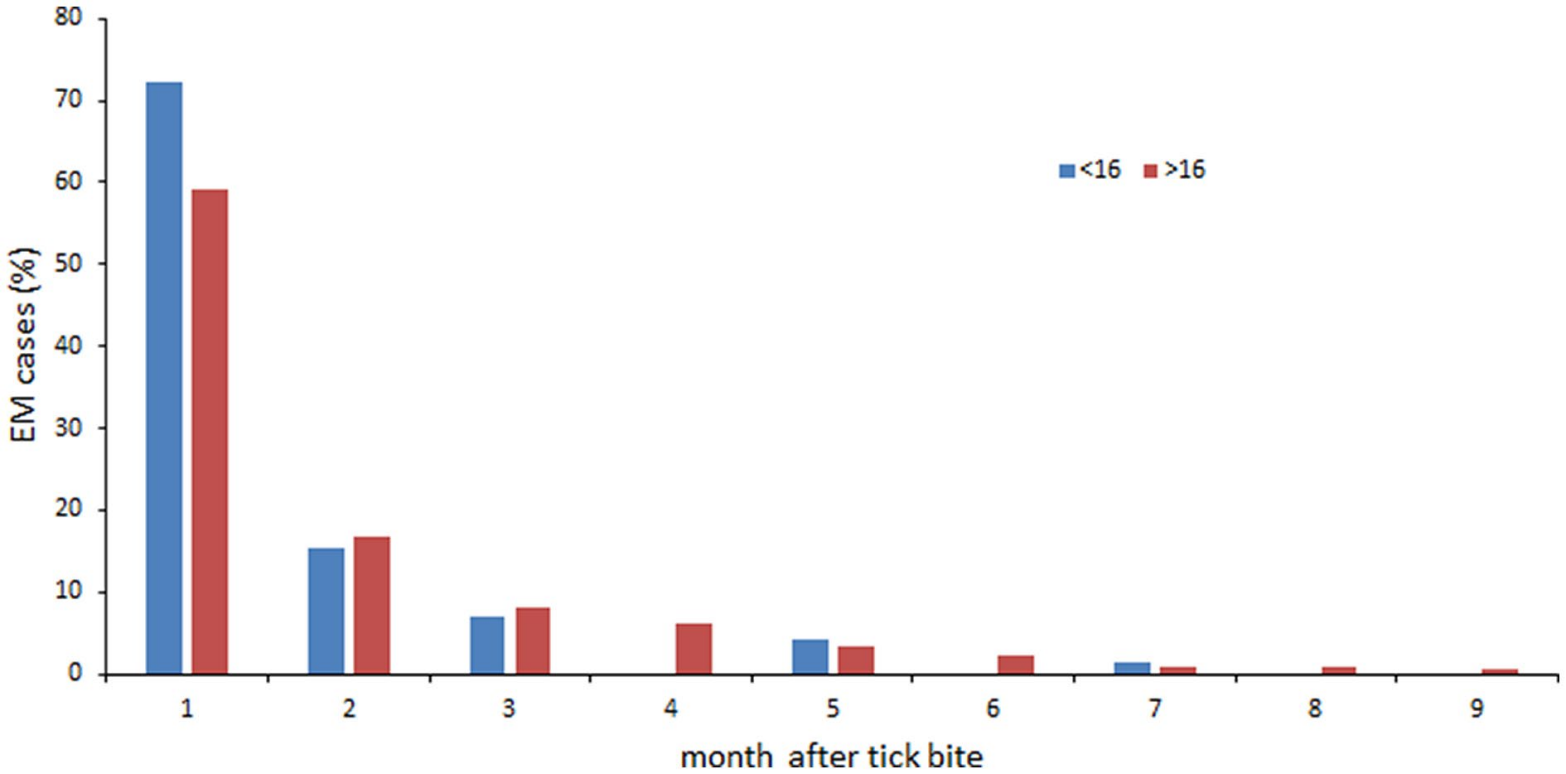
Delay of appearance of confirmed EM. <16 years: blue bars; >16 years: orange bars.

Furthermore, we analyzed their delay of appearance after tick bite (Figure 9). The great majority of EM were detected during the first month after tick bite (60.2%, *p* < 0.00001) followed by the second month (16.5%) and the third month (8.4%). When we compared children and adults, a statistical difference is observed during the first month where the prevalence in children is superior to that of adults (*p* = 0.03). We did not see any difference during the following months at the exception of the 4th month for which the number of cases in children was inferior to adults (*p* = 0.03).

The size of the EM was further studied. Most of the EM (48.9%) had a size between 5 and 10 cm. Only 10% of the EM had a size over 15 cm. We then investigated whether a correlation could exist between the size of the EM and the presence/absence of systemic symptoms. Our results show that such a correlation does not exist.

##### Early disseminated phase symptoms

We then analyzed the symptoms observed during the early disseminated phase of Lyme borreliosis that constituted 20% of the reported cases (Supplementary Table S3).

###### Neurological symptoms

We observed a majority of neuroborreliosis (57.8%) alone or accompanied with EM (38.3%) or cardiac signs (AVB: 0.4%) or ocular signs (2.4%). Facial paralysis alone or accompanied with other signs accounted for 55.6% of the cases. Meningoradiculoneuritis and radiculitis were observed in 27% of the patients and meningitis in 10.9% of the cases. We never observed any arthritis accompanying neurological symptoms. Interestingly, neurological signs were more frequently observed in male (65.7%) than in female (34.3%) (*p* < 0.00001).

We first analyzed whether a difference of prevalence could be observed over the years of study. We found out that the percentage of neuroborreliosis was statistically lower in 2005 and 2007 compared to the other years (*p* < 0.00001).

We then analyzed if there was a difference between the prevalence of neurological symptoms according to the age groups. The number of neurological cases were clearly higher in class <16 years than that recorded in the other classes of age (*p* < 0.00001).

**Figure 10:**
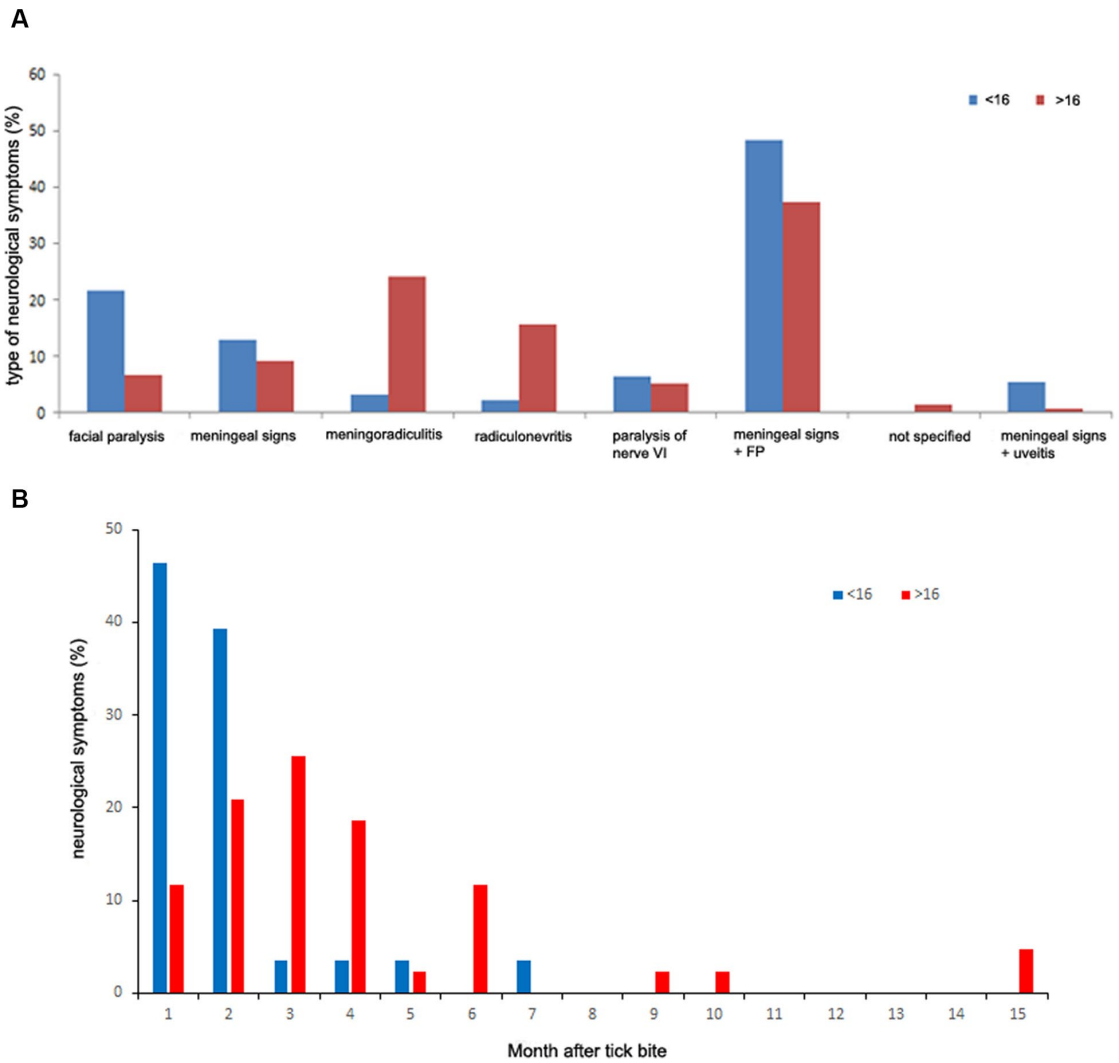
Neurological symptoms characterization. **(A)** Neurological case presentation as function of age. **(B)** Delay of appearance as function of age: blue bars: <16 years; orange bars: >16 years.

We also verified whether there was a difference in the neurological case presentation as function of age. For age class <16 years, a significant difference appears with meningeal signs accompanied by facial paralysis that are preferentially observed (*p* < 0.00001). In adults, the meningeal signs accompanied by facial paralysis and meningoradiculitis symptoms were more prevalent than the other signs (*p* < 0.00001) (Figure 10A). Now if we compare children and adults, facial paralysis and meningeal signs with uveitis were more often observed in children than in adults (*p* = 0.009 and *p* = 0.02 respectively). meningoradiculitis and radiculitis more often in adults (*p* < 0.00001). No difference was found for meningeal signs alone or accompanied by facial paralysis between adults and children.

We also investigated the delay of appearance of the neurological symptoms. Neurological signs appeared mainly during the 4 months following the tick bite. We did not find any statistical difference between these months.

We verified whether the age of the patient could modulate the delay of apparition of these symptoms. For the first month, the number of neurological signs is much lower in adults (11.6%) than in children (46.4%) (*p* < 0.001). The second month shows no difference between adults and children. For the third month the difference is reversed in favor of adults (25.6%) versus 3.6% for children (*p* = 0.015) (Figure 10B).

###### Articular manifestations

They accounted for 24.9% of the confirmed cases of the early-disseminated phase. They consisted in acute arthritis. Lyme arthritis was mainly observed alone (92.5%) or less frequently accompanied by EM, uveitis or neuroborreliosis (Supplementary Table S4). Monoarthritis (67.2%) was predominant. Acute arthritis was observed to be more frequent in male (65.7%) than in female (34.3%) (*p* < 0.00001). Adults (78%) were more affected than children (22%) (*p* < 0.00001). We also analyzed the prevalence of Lyme arthritis over the years of study. We observed a significantly lower number of rheumatoid cases in 2003, 2004, and 2005 (*p* < 0.00001).

###### Other symptoms

The other symptoms of the early disseminated phase were BL (6.8%), cardiac manifestations (4.9%) and ocular symptoms (5.6%) (Supplementary Table S5). BL occurred more frequently in male (72. 4%) than in female (27.6%) (*p* < 0.0001) as well as in adults (92.8%) compared to children (7.2%) (*p* < 0.00001). Atrioventricular block was the main cardiac manifestation. There was no sex bias for this particular manifestation.

##### Late disseminated phase

In the late disseminated phase (3.4%), there was a majority of ACA (50.7%) more frequently reported in lower limbs (86.9%) than in upper limbs (13.1%) (*p* < 0.0001), as well as in female (69.4%) compared to male (30.6%) (*p* = 0.001). It was never observed in children (*p* < 0.00001).

Chronic arthritis (43.7%) and late neurological signs (5.6%) were also described (Supplementary Table S5).

#### Serology of the confirmed cases

67.6% of the confirmed cases had a demand for a serology (Table 3). As shown in Table 3, 100% of the serology not done consisted in EM.

**Table 3:** Serological tests performed on the confirmed cases.

Serology type	% cases (%EM)
Not done	32.4 (100)
Positive skin biopsy, or positive joint fluid	0.7 (6.7)
Negative or doubtful serology	12 (99.2)
Positive serology (WB results not reported)	27.6 (89.2)
Positive serology, positive WB (IgM and/or IgG)	14.3 (16.6)
Positive serology, positive CSF ITS not done + EM	1.3 (92.6)
Positive serology, positive CSF, lymphocytosis or ITS made	11.3
Positive serology, positive CSF ITS not done	0.05
Positive serology (cross reaction with syphilis or auto-immune disease)	0.01
Not specified	0.4

#### Treatment

**Figure 11:**
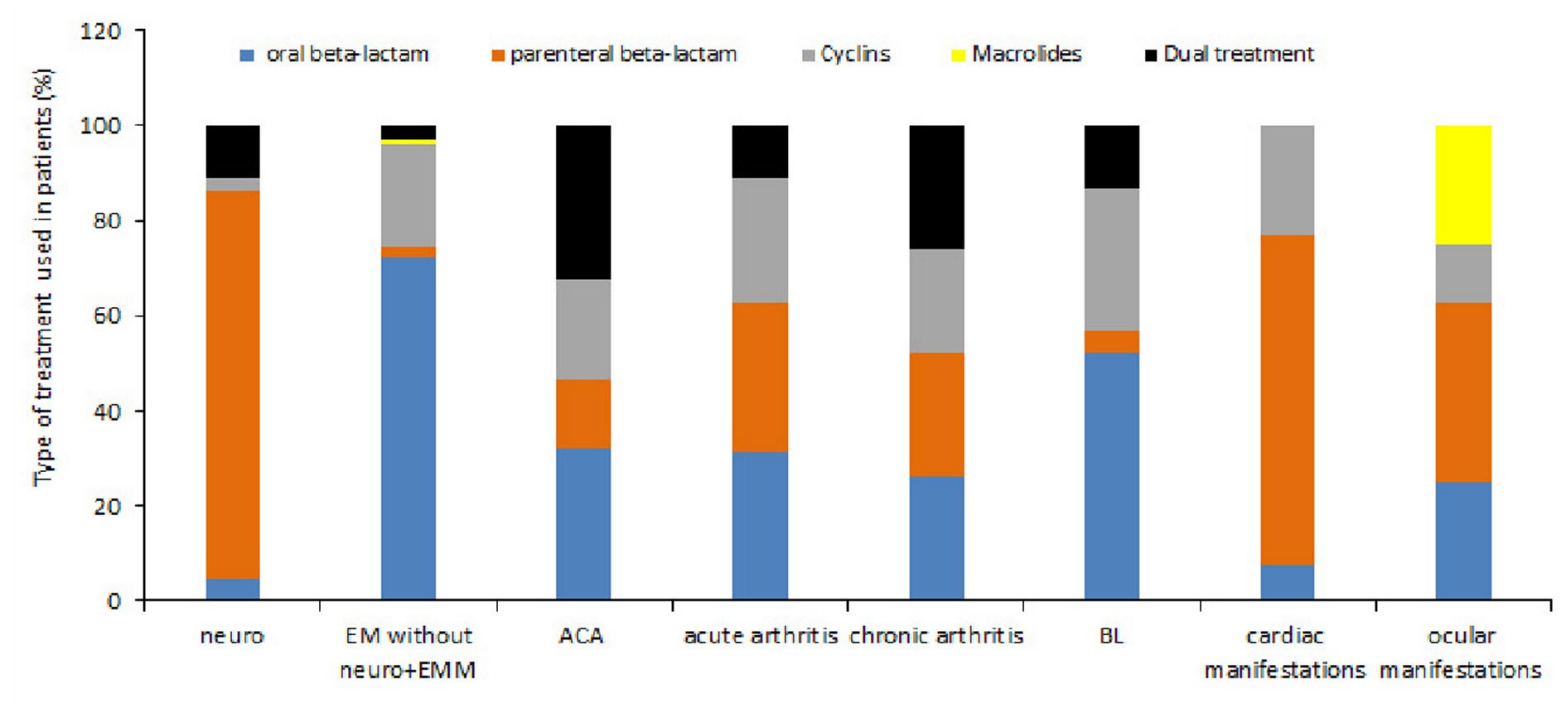
Type of treatment used according to the various symptoms observed. The type of treatment is presented as function of colors (oral beta-lactam: blue, parenteral beta-lactam: orange, cyclins: gray, macrolides: yellow and association or succession of antibiotics: black) and according to the symptoms observed.

We investigated the various treatments used according to the various case definitions taking into account only cases where this notion was indicated (Figure 11). For EM, oral beta-lactam was principally utilized (71.7%) whereas for neurological symptoms parenteral beta-lactam was used (89.9%). Arthritis was treated with either oral or parenteral beta lactam or cyclins in equivalent proportion while cardiac symptoms were essentially treated with parenteral beta-lactam (62.5%). The administration of two antibiotics (dual serial treatment) has been used primarily for patients with ACA or arthritis.

We studied the duration of the different treatments according to the reported symptoms (Table 4). We can see that oral beta-lactam was prescribed for about 3 weeks whatever the symptom if it was unique. However, we observe a longer administration in case of associated symptoms. Parenteral beta-lactam were used for an average of 3 weeks. A longer duration was observed in case of ocular symptoms. Cyclins were administered for an average of 1 month except for EM, cardiac symptoms or associated symptoms where a duration of 3 weeks was observed. The duration of administration of two antibiotics was variable, between 15 and 30 days depending on the symptomatology, which is in accordance with the recommendations of the French National Authority for Health.

**Table 4:** Treatment time according to the clinical manifestations recorded.

Symptoms	Oral beta-lactam (day)	Parenteral beta-lactam (day)	Cyclin (day)	Macrolide (day)	Dual treatment (day)
EM	16.3 ± 5.3	18.6 ± 4.5	17.1 ± 6.9	18.0 ± 10.2	16.9 ± 6.5
BL	17.7 ± 6.1		34.6 ± 18.1		21
MEM	18.2 ± 4.4				30.0 ± 12.7
ACA	23.7 ± 4.2	21	27.8 ± 5.2		25.7 ± 12.8
Neurological	19.0 ± 3.5	22.5 ± 3.6			12.6 ± 5.3
Rheumatological	25.2 ± 15.5	20.2 ± 4.1	30.1 ± 16.1		23.5 ± 12.8
Cardiac	21	19.8 ± 5.6	18.0 ± 4.2		
Ocular	21	25.5 ± 6.4	30	22.5 ± 2.1	
Dermatological + neurological	27.0 ± 3.5	21.5 ± 3.8	21.3 ± 1		15.4 ± 8.6
Dermatological + rheumatological	38.0 ± 32.5		21		15
Dermatological + cardiac			21		

The duration is shown as the mean ± standard deviation.

### Probable cases

Probable cases represented 10% of the reported cases (350). The average age of the population was 47 [2–89]. Seven percent of the subjects were at risk due to exposure to professional risks. The other patients were exposed to tick bite during their leisure (95%) (*p* < 0.00001).

Nonspecific systemic symptoms were observed in 35.2% of the patients. Dermatological signs (44.6%) consisted in 91% possible EM cases. The EMs were observed alone in 34% or were accompanied by systemic nonspecific signs (66%).

The early disseminated phase (51.1%) showed a majority of neurological signs (83.8%) (*p* < 0.00001). They were more often reported in children (71.4%) than in adults (39.3%) (*p* < 0.00001). The major neurological manifestation in children was facial paralysis that was more prevalent than meningitis and meningoradiculitis (*p* < 0.00001) Prevalence of facial paralysis was significantly higher in children (*p* < 0.00001) whereas meningoradiculitis and radiculitis were significantly more reported in adults (*p* = 0.004 and *p* < 0.0001 respectively). Meningeal signs were the less prevalent symptom in adults (*p* < 0.00001).

Arthritis (11.7%) was observed preferentially in adults (95%) (*p* < 0.00001). The late disseminated phase was observed in 8.3% of the cases and consisted in ACA [41.4%, never reported in children (*p* < 0.00001)], neurological signs (34.5%) and chronic arthritis (24.1%).

All of the 350 probable cases received a treatment. Among the 160 neurological cases treated, 78.6% received parenteral beta-lactam and 10.7% a dual treatment. 71.7% of the EM received oral beta-lactam. Patients with acute arthritis were treated either by cyclins (58.3%), oral beta-lactam (25%) or parenteral beta-lactam (8.3%).

### Doubtful cases

Doubtful cases represent 13.5% of the reported cases. They were more often reported in adults (85.1%) than in children (14.9%) (*p* < 0.00001). 57.3% of the patients alleged a tick bite before consultation.

When we look at the repartition of dermatological cases (20%) between children and adults, we see that the prevalence in children is inferior to that of adults (*p* < 0.00001) (20.4 and 79.6% respectively).

Neurological symptoms (66%) were mainly observed (p < 0.00001) and more often reported in adults than in children (85.2 and 14.8% respectively) (*p* < 0.00001). The major neurological manifestation in children was facial paralysis (75.6%) that was more often reported than the other signs (*p* < 0.00001). In adults, radiculitis was the most prevalent symptom (53.5%) (*p* < 0.00001).

Arthritis represented 8% of the doubtful cases and were more often reported in adults (92.1%) than in children (7.9%) (*p* < 0.00001).

Among the 474 doubtful cases, 80% got a treatment. 70% of the dermatological cases received the administration of beta-lactam by the oral route. Among the neurological cases treated, 57% received parenteral beta-lactam, 20% received oral beta-lactam and 11% had a dual treatment. Arthritis was either treated by oral beta-lactam (34.4%), 21.9% received parenteral beta-lactam, cyclins were used in 28.1% of the reported cases and 15.6% received a dual treatment.

### Asymptomatic seropositive cases

They represented 10.2% of the reported cases (358). There was a gender bias, men being more numerous than females (*p* = 0.011). The average age of the study population was 51 ± 20, statistically superior to the age of the other case classifications. They were more often reported in adults (93.4%) than in children (6.6%) (*p* < 0.00001). When exposure to risk was known, it was recreational for 76.7% of the patients and occupational for 21.6% of them. 50.5% of the patients reported a tick bite before consultation with the doctor.

Sixty-six percent of the patients presented nonspecific systemic symptoms: 43.2% had arthralgia with or without symptoms, 36.3% had other systemic symptoms (asthenia, myalgia) with or without tick bite.15.4% had other symptoms (fall, cognitive disorders, gout, HIV, streptococcal endocarditis, etc.). 7.5% had a history of EM and 8.7% had neurological signs (stroke, Guillain Barré). Articular signs represented 6.7% of the cases. No ACA was reported. Of these 358 cases, 60.1% were treated. The treatment given was preferentially oral beta-lactam (43.2%) followed by parenteral beta-lactam (24.3%) and 20.4% cyclins (*p* < 0.00001).

### Negative cases

Two hundred and twenty-three negative cases of LB (6.4%) were identified among the patients file. The average age of the patients was lower than in the other cases: 43 ± 22 compared to 49 ± 20.7 for the confirmed cases. 97.8% of the patients were exposed to recreational risks that could explain why they were treated by doctors as LB. A tick bite before the current symptoms was reported in 76.2% of the patients. The symptoms reported were the following: 21.1% neurological symptoms, 1.8% cardiac symptoms, 3.6% articular symptoms, 16.1% dermatological symptoms, 15.2% systemic symptoms (18% arthralgia) with or without tick bite, 0.9% ACA and 15.7% had no symptoms but had a tick bite. These patients had a negative serology or it was not performed. Only 41.7% of the patients received a treatment. The treatment given was preferentially beta-lactam either oral (50.6%) or parenteral (27.6%) followed by cyclins (15%) then macrolides (1.14%) (*p* < 0.0000).

## Discussion

In order to study the epidemiology of LB in France, we contacted general practitioners, specialists and clinicians to collect data on suspected cases of LB. We collected information on 3,509 cases, which were classified according to the current recommendations on the diagnosis of Lyme borreliosis. An app was designed in order to visualize all the results on maps and sanky plots^1^. We used for the first time in LB study, reduction dimension techniques also known as MCA as well as hypothesis testing to analyze the patients’ profiles to discern good markers that discriminate their heterogeneity. Interestingly, the MCA, which is an unsupervised statistical method, showed a robust segregation of case definition in good agreement with the structure of cutaneous, neurological, cardiac, rheumatologic, and ocular cases. In particular, it allowed identifying three principal clusters of patients: one cluster of asymptomatic seropositive and negative patients, and two clusters of confirmed, probable and doubtful patients. Using the quality measures of the MCA, we were able to select the variables that characterize the structure of these clusters. It turns out that symptoms, with neurological and dermatological cases explaining the split of confirmed/probable/doubtful cases into two clusters, as well as treatments and serological status are key parameters to explain the variability between these clusters. Visualization and quality measures suggest that the age of patient is a factor explaining the substructure within these three clusters. This result is harder to investigate because of the rather low number of children compared to that of adults.

Several regions of France, Auvergne-Rhône-Alpes, Grand-Est, Bretagne, Normandie and Ile-de-France, were identified as more at risk than others. This is in agreement with a recent study, which showed that main spatial clusters of LB cases were reported in central and northeastern France every year between 2016 and 2019 (23). While we cannot exclude the possibility of bias due to the uneven distribution of doctors willing to participate to this survey, tick collection studies carried out in the various regions mentioned showed a high rate of infection of ticks with *Borrelia burgdorferi* sensu lato (Elisabeth Ferquel personal communication), which is in agreement with our observations. In the regions and departments that we systematically studied, we found that the number of reported cases fluctuated over time. Remarkably, the reported clinical manifestations also fluctuated within the years of study. Such observations have already been reported in the literature by Peney et al. (24).

We studied more in detail the characteristics of the 3,509 reported cases. The mean age of the population is 48 years. We were able to specify a difference in age according to the case definition we established. Asymptomatic seropositive cases had a higher average age than negative cases. As in other studies, we observed a bimodal distribution of cases according to age groups with two peaks, one being children aged 6 to 10 years and the other adults aged 55–60 years. We observed a difference in gender in classes 6–15 and 41–45 years, males were more numerous than females, whereas it is the opposite for classes 51–60 and 86–90 years. The fact that in children, boys outnumbered girls has already been described in other studies (25). We also showed that there was a difference in gender according to the case definition. This observation that women were more numerous in confirmed cases of LB had previously been reported in other studies (26). We also analyzed the risks of exposure to tick bites, both occupational and recreational, for which we obtained a response in our survey. Among the group with occupational risks, farmers were the most exposed, as already shown in other studies (27). Most of the remaining patients were engaged in some nature-related activity, which has been reported in other studies (28). Contrary to what we observed for occupational risks where asymptomatic seropositive cases were the most numerous, confirmed cases had a greater risk of exposure to recreational risks than asymptomatic seropositive cases (29).

Our questionnaire also required to specify the notion of tick bites, either in the months/years before the consultation (previous EM/EM history), or in direct relation to the consultation. We were able to show that more than a third of the patients had already had one or more tick bites in their life, which showed that they were naturally exposed. 69.4% of them had observed a tick bite shortly before the consultation. This figure is quite consistent with studies conducted in Europe on patients with EM (30, 31). We observed that the notion of a tick bite having preceded the diagnosis was higher in the case of EM (79.5%) whereas it was statistically lower in the case of symptoms of the disseminated phase (54.4%). The Sentinel network observed it in 2020 (32). While for children, the majority of bites were located on the head, upper limbs and trunk, they were generally located on the lower limbs for adults, as already reported (31).

We observed a seasonal distribution of the LB suspected cases reported in this study, which is in good agreement with the seasonality of tick activity. The vast majority of bites occurred from May to September while diagnoses were made from June to December. Although until 2008 there had been a decrease in the number of diagnoses during winter, a trend seemed to appear from 2008 onwards where the number of diagnoses remained high even during the winter months, probably explained by the climate change and winters being wet and milder, extending thereby the tick activity as well as human outdoor activities (33).

At the time of diagnosis, general non-specific symptoms of LB were observed in 32.7% of patients, which is lower to that reported by Santino and Longobardi (34) who described 41% of LB suspected cases presenting systemic symptoms. These manifestations can be explained by others pathogens also transmitted by ticks (12). MCA showed that general symptoms tended to overlap with negative or asymptomatic seropositive individuals. Interestingly, in our study, there was a clear correlation between the prevalence of headache and fever and the occurrence of neuroborreliosis.

Among the Lyme specific symptoms reported, skin manifestations were the most frequent (62.6%), followed by neurological manifestations (26%), joint manifestations (7%) and finally cardiac and ocular manifestations (less than 2% for each).

As expected, the most frequent skin manifestation was EM (89.4%). In most of the confirmed EM cases (73%), we did not observe any general symptoms. In the US, 69% of EMs were accompanied by systemic symptoms, whereas in Europe it has been reported that 38% of cases had systemic symptoms (3). Our figures were slightly lower (23.7%). 79.2% of patients with EM recalled having had a tick bite, which is in the high range of data observed in Europe (33). As previously reported (31), females were more affected than men. We also observed that EMs were statistically more frequent in adults than in children. The vast majority of EMs occurred within 2 months of the tick bite. Six percent of EMs were accompanied by neuroborreliosis. Although rare, these dual clinical manifestations were statistically much more common in adults than in children. EM was less often accompanied by arthritis or Lyme carditis (0.2%). MEM were rarely observed in our study (0.9%).

If we now look at the diagnosis of confirmed EM without associated symptoms, a serology was not requested for 45.1% of the cases and if requested was positive in 69.9% of cases. This figure is quite different from what was reported by Charbonneau et al. in Canada (35) who showed that 66% of patients with EM had a negative serology (35). Our results were supported by the MCA where EM matches either with serology not done, positive, negative or doubtful. Regarding the treatment of EM, the vast majority of cases received oral antibiotics (91.7%). Interestingly, a small number of patients received parenteral betalactam (3.8%) in the absence of any other symptoms.

ACA and BL were rarely reported (respectively 1.7 and 1.4% the confirmed cases) and while ACA was more frequent in females (69.4%), BL was more often reported in male (72.4%) as previously shown (36). However, we did not find any case of ACA in children, which is very rare in children (37) as well as more cases of BL in children than in adults as reported in other studies (38).

*Borrelia valaisiana* and *Borrelia afzelii* have been identified in skin biopsies from ACA in our study. Although it is rarer to find *Borrelia valaisiana* in skin biopsies, this bacterium has been found by PCR in skin biopsies and in cerebrospinal fluid from a patient with neuroborreliosis (4).

Neurological manifestations accounted for 23.3% of the reported cases. We found a higher prevalence of neuroborreliosis in males (65.9%) than in females (34.1%), as previously reported (39) and in children (39%) compared to adults (8.8%). The latter observation may be explained by the fact that children are more easily bitten on the head and upper limbs, as observed in this study, and also by the hypothesis that the bacteria could progress along the nerve roots (40). The clinical presentation was not the same between children and adults. Adults were more likely to have radiculitis or meningoradiculitis, whereas children were more likely to have facial paralysis with meningeal signs as observed in European children affected by early neuroborreliosis (41–44). These symptoms appeared very quickly, in the first 2 months after the bite in children, whereas in adults they appeared from the second month on with a peak in the third month. If we now consider the diagnosis of the confirmed neurological cases, more than half (43.3%) had an ITS while in 46% of the cases we note the absence of ITS but the presence of lymphocytosis and hyperproteinorachia. In probable neurological cases, although symptoms suggestive of neuroborreliosis were present, CSF was positive but the presence of lymphocytosis and hyperproteinorachia were not available, unlike in confirmed cases. Indeed, MCA hardly identified any difference between a sub cluster of confirmed and probable cases, as the difference relies in the type of serology performed. Less than 5% received oral doxycycline, a treatment which was shown as efficient as parenteral beta-lactam in the treatment of neuroborreliosis (45, 46).

Lyme specific articular manifestations (6.6%) consisted mainly in acute arthritis (77.5%). Men (66.2%) were statistically more affected than women, as has already been described in the literature (36). We report one case of isolation of *Borrelia afzelii* from joint fluid, a borrelial species that had previously been described in cases of Lyme arthritis (47). Patients with arthritis were treated with either oral beta-lactam, parenteral beta-lactam or cyclins in equal proportions.

Lyme cardiac and ocular manifestations were rarer and each reached about 1% of the recorded cases as previously reported in other studies (19, 48, 49). Lyme carditis manifested itself mainly as atrioventricular block (52.4%), as has already been specified in the literature (50, 51). It was not observed in children in our study. We also did not observe a gender bias as reported in other studies where men were more affected than women (52), possibly because of the relative low number of patients that were identified. Patients were treated primarily with parenteral beta-lactam. Confirmed ocular cases were mainly uveitis (87.5%) and keratitis (12.5%). In children, these manifestations were always accompanied by neurological symptoms. Patients received either parenteral or oral beta-lactam or macrolide.

A good correlation was found between the definition of the cases and the percentage of treated cases. As expected, doubtful cases were more often treated (80%) than asymptomatic seropositive cases (60%) or negative cases (41.7%). We focused on asymptomatic seropositive cases. These patients had already been in contact with the bacteria either during confirmed infections or due to asymptomatic seroconversion. Interestingly, 21.6% of them had professions at risk of exposure to tick bite (farmers, foresters, etc.). The analysis of the patients’ symptoms showed that a large proportion of them (66.1%) presented general non-specific symptoms. We were not able to obtain more details about possible previous episodes of Lyme borreliosis for those patients. We have no knowledge of previous treatment. It is therefore difficult to consider those cases as chronic LB, although several studies mention this possibility (53).

In conclusion, it is the first time that a comprehensive study of supposed Lyme borreliosis cases has been conducted over several years in France. This study identified a set of variables that were used and validated in the MCA. We used a very stringent case definition, showing a number of probable cases that were most likely LB cases. As expected, the predominant clinical presentation was EM but other specific manifestations of borreliosis like neuroborreliosis and Lyme arthritis were also present in proportion previously described in France (12), higher than in Germany (26) but similar to other studies in Europe (34). Our studies confirm that children have a distinct clinical presentation from adults. This is also a consequence of the different location of the tick bite in children, which as reported also by other authors is often on the head (54, 55). We also observed that there is a gender effect on clinical presentation, with females presenting more often with EM or ACA than males, while males present more often with neurological signs or arthritis than females. Hypotheses have been raised to try to explain these differences (56) but further studies should be carried out to better understand these observations (57). A limitation of this comprehensive study is that we were not able to follow the clinical course of patients after treatment, particularly in cases that we defined as asymptomatic seropositive or negative for LB. All these results suggest the interest of refining the questionnaire to avoid missing data and of following up a cohort of patients over a sufficiently long period to obtain more data on their fate according to different parameters (case definition, symptoms, treatment).

## Data availability statement

The raw data supporting the conclusions of this article will be made available by the authors, without undue reservation.

## Ethics statement

Ethical approval was not required for the study involving humans in accordance with the local legislation and institutional requirements. Written informed consent to participate in this study was not required from the participants or the participants’ legal guardians/next of kin in accordance with the national legislation and the institutional requirements. Written informed consent was not obtained from the individual(s) for the publication of any potentially identifiable images or data included in this article because the general practitioner was in charge to receive the oral consent of the patient for the use of the data by the NRC.

## Author contributions

EP: Data curation, Formal analysis, Methodology, Software, Writing – original draft, Writing – review & editing. LC: Data curation, Formal analysis, Methodology, Writing – review & editing. J-CG: Formal analysis, Investigation, Methodology, Validation, Writing – review & editing. MV: Writing – review & editing. EF: Conceptualization, Formal analysis, Funding acquisition, Investigation, Methodology, Project administration, Validation, Writing – original draft, Writing – review & editing, Data curation, Supervision. VC: Conceptualization, Formal analysis, Funding acquisition, Investigation, Methodology, Project administration, Resources, Validation, Writing – original draft, Writing – review & editing.

## Footnotes

^1^ https://lymeborreliosis.shinyapps.io/ShinyApp/
