# Supplementary material for: Case presentation and management of Lyme disease patients: a 9-year retrospective analysis in France
Source: Front Med (Lausanne). 2024 Jan 17;10:1296486. doi: 10.3389/fmed.2023.1296486 (PMC10829333; doi:10.3389/fmed.2023.1296486)
Supplement: Supplementary file 1 [file Presentation_1.pptx]

## Slide 1
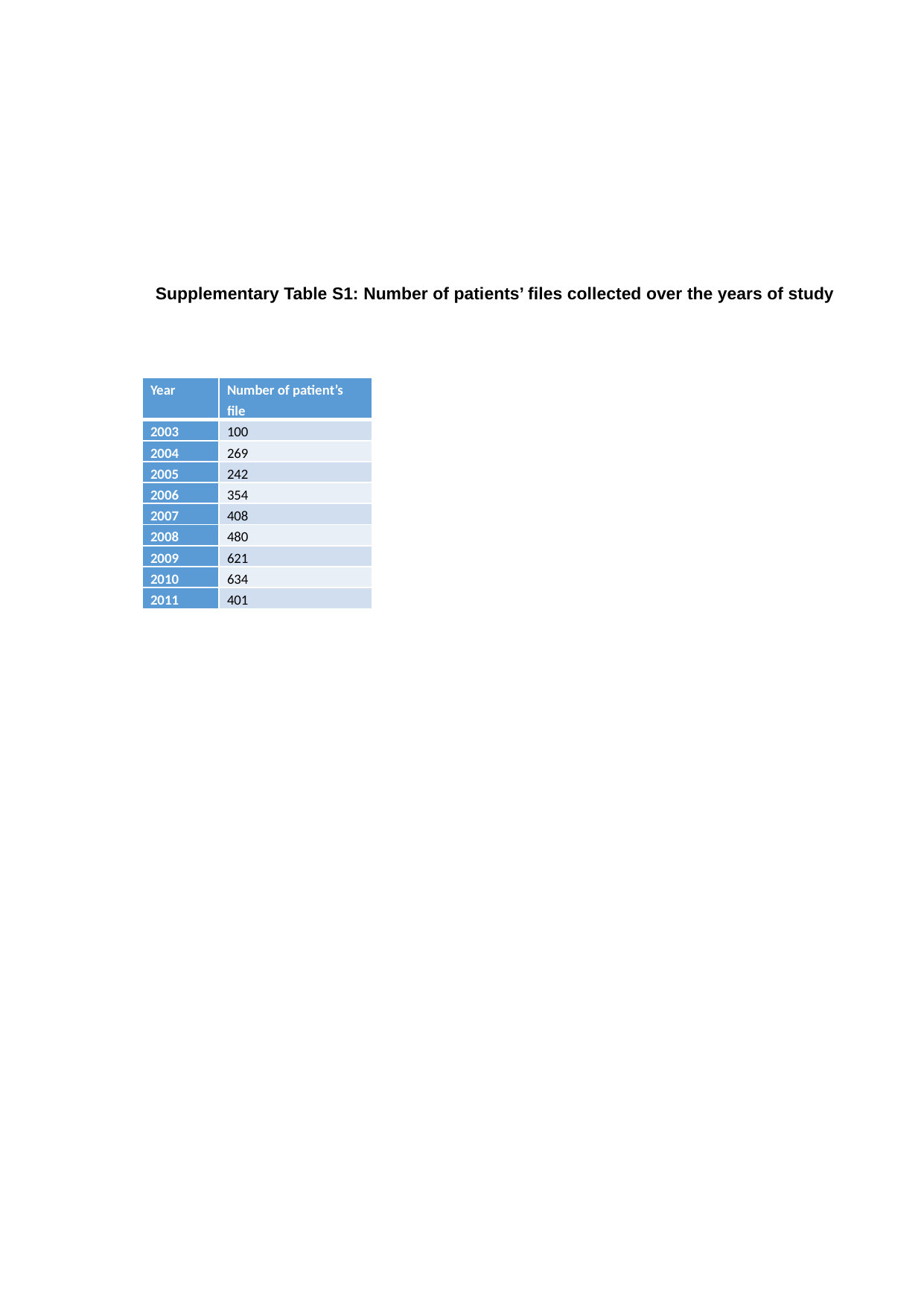

Supplementary Table S1: Number of patients’ files collected over the years of study
| Year | Number of patient’s file |
| --- | --- |
| 2003 | 100 |
| 2004 | 269 |
| 2005 | 242 |
| 2006 | 354 |
| 2007 | 408 |
| 2008 | 480 |
| 2009 | 621 |
| 2010 | 634 |
| 2011 | 401 |

## Slide 2
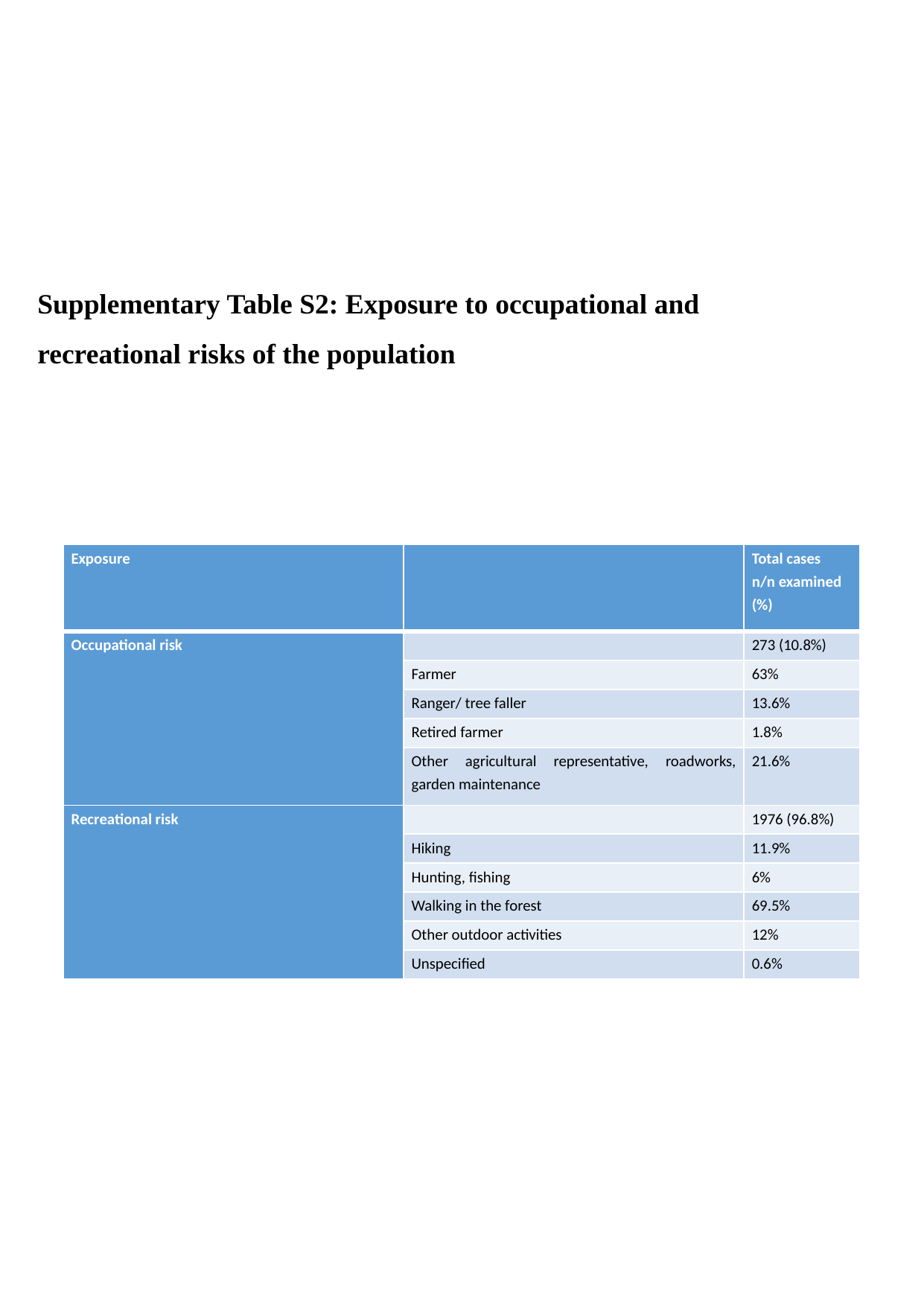

Supplementary Table S2: Exposure to occupational and recreational risks of the population
| Exposure | | Total cases n/n examined (%) |
| --- | --- | --- |
| Occupational risk | | 273 (10.8%) |
| | Farmer | 63% |
| | Ranger/ tree faller | 13.6% |
| | Retired farmer | 1.8% |
| | Other agricultural representative, roadworks, garden maintenance | 21.6% |
| Recreational risk | | 1976 (96.8%) |
| | Hiking | 11.9% |
| | Hunting, fishing | 6% |
| | Walking in the forest | 69.5% |
| | Other outdoor activities | 12% |
| | Unspecified | 0.6% |

## Slide 3
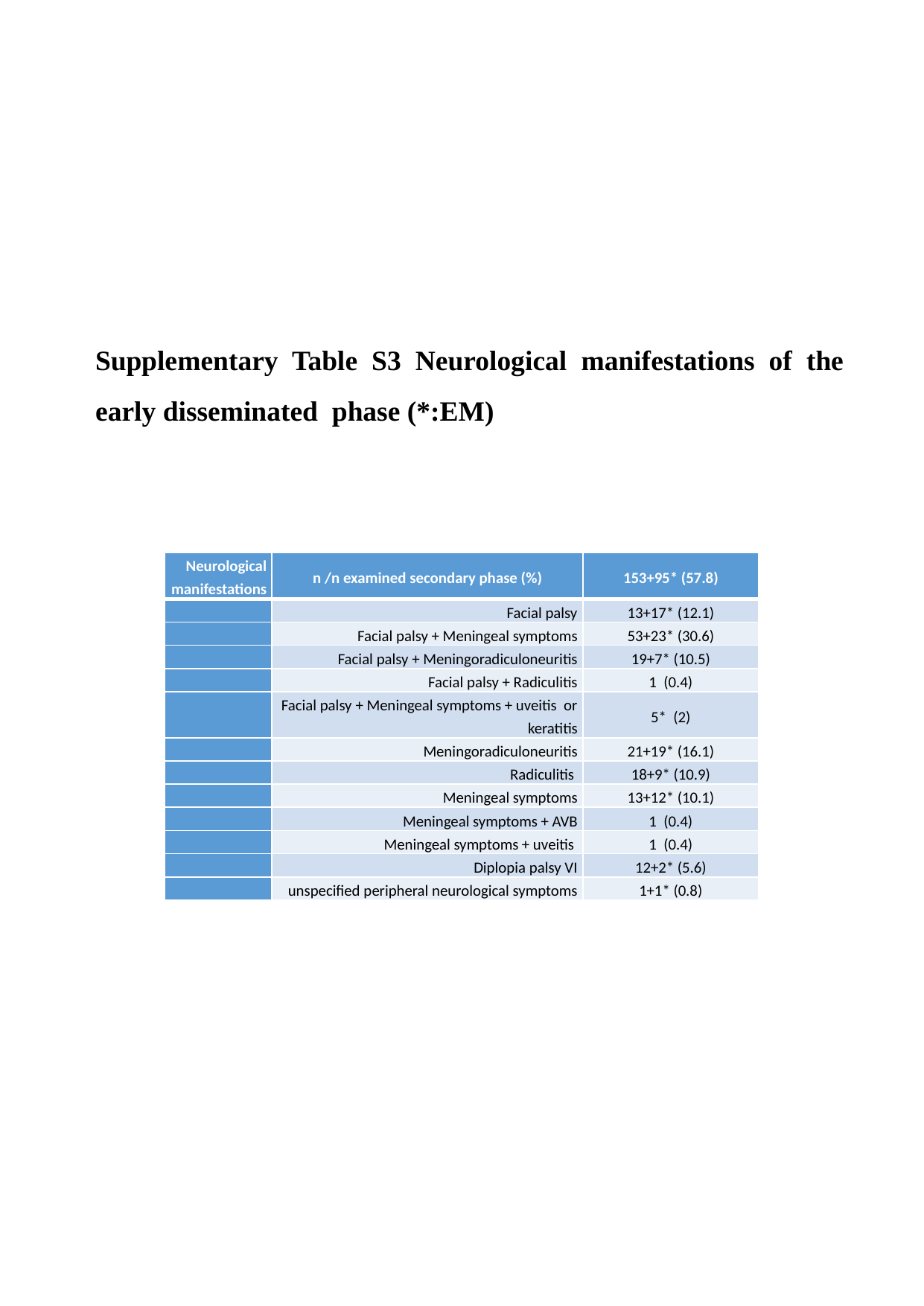

Supplementary Table S3 Neurological manifestations of the early disseminated phase (*:EM)
| Neurological manifestations | n /n examined secondary phase (%) | 153+95\* (57.8) |
| --- | --- | --- |
| | Facial palsy | 13+17\* (12.1) |
| | Facial palsy + Meningeal symptoms | 53+23\* (30.6) |
| | Facial palsy + Meningoradiculoneuritis | 19+7\* (10.5) |
| | Facial palsy + Radiculitis | 1 (0.4) |
| | Facial palsy + Meningeal symptoms + uveitis or keratitis | 5\* (2) |
| | Meningoradiculoneuritis | 21+19\* (16.1) |
| | Radiculitis | 18+9\* (10.9) |
| | Meningeal symptoms | 13+12\* (10.1) |
| | Meningeal symptoms + AVB | 1 (0.4) |
| | Meningeal symptoms + uveitis | 1 (0.4) |
| | Diplopia palsy VI | 12+2\* (5.6) |
| | unspecified peripheral neurological symptoms | 1+1\* (0.8) |

## Slide 4
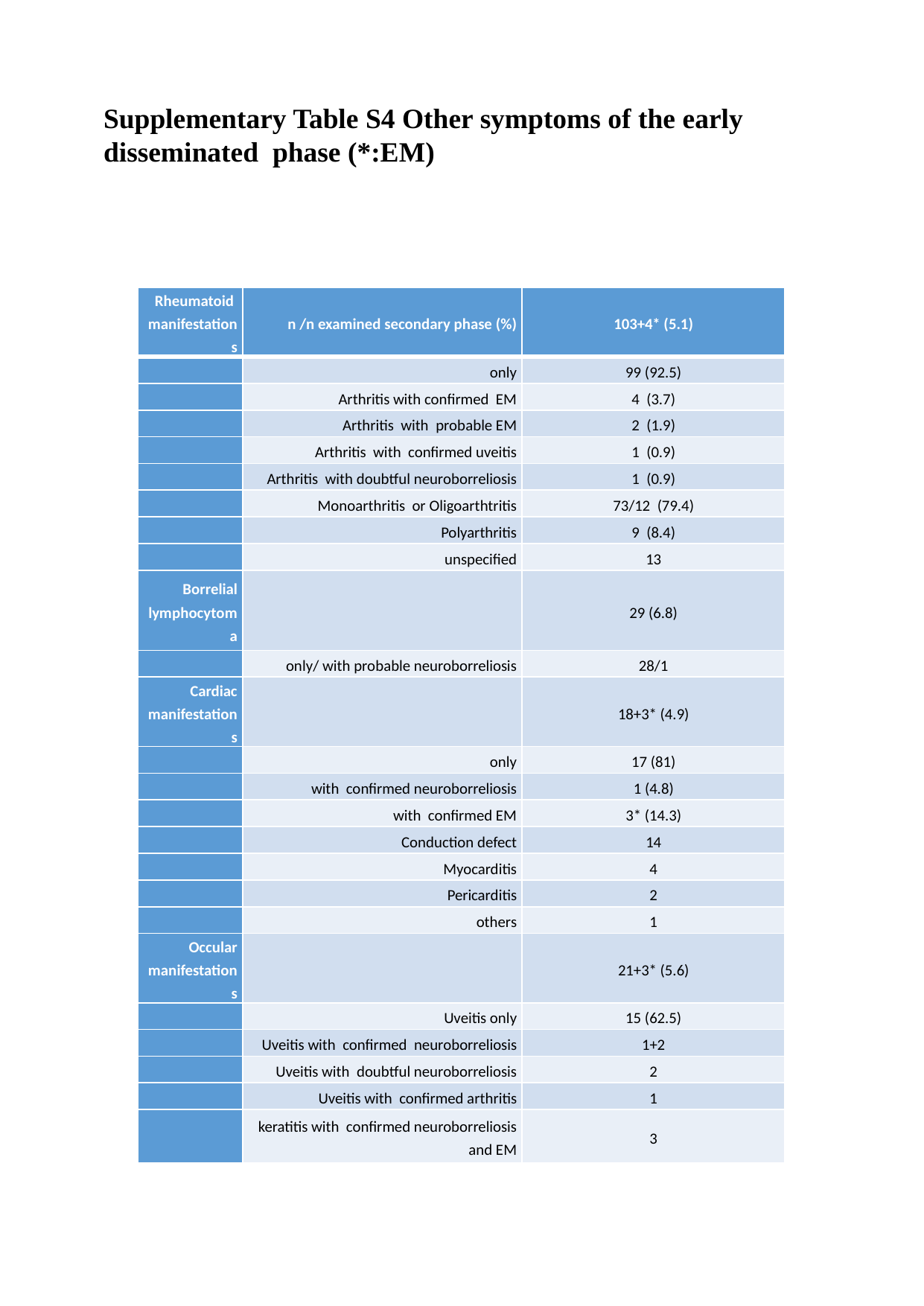

Supplementary Table S4 Other symptoms of the early disseminated phase (*:EM)
| Rheumatoid manifestations | n /n examined secondary phase (%) | 103+4\* (5.1) |
| --- | --- | --- |
| | only | 99 (92.5) |
| | Arthritis with confirmed EM | 4 (3.7) |
| | Arthritis with probable EM | 2 (1.9) |
| | Arthritis with confirmed uveitis | 1 (0.9) |
| | Arthritis with doubtful neuroborreliosis | 1 (0.9) |
| | Monoarthritis or Oligoarthtritis | 73/12 (79.4) |
| | Polyarthritis | 9 (8.4) |
| | unspecified | 13 |
| Borrelial lymphocytoma | | 29 (6.8) |
| | only/ with probable neuroborreliosis | 28/1 |
| Cardiac manifestations | | 18+3\* (4.9) |
| | only | 17 (81) |
| | with confirmed neuroborreliosis | 1 (4.8) |
| | with confirmed EM | 3\* (14.3) |
| | Conduction defect | 14 |
| | Myocarditis | 4 |
| | Pericarditis | 2 |
| | others | 1 |
| Occular manifestations | | 21+3\* (5.6) |
| | Uveitis only | 15 (62.5) |
| | Uveitis with confirmed neuroborreliosis | 1+2 |
| | Uveitis with doubtful neuroborreliosis | 2 |
| | Uveitis with confirmed arthritis | 1 |
| | keratitis with confirmed neuroborreliosis and EM | 3 |

## Slide 5
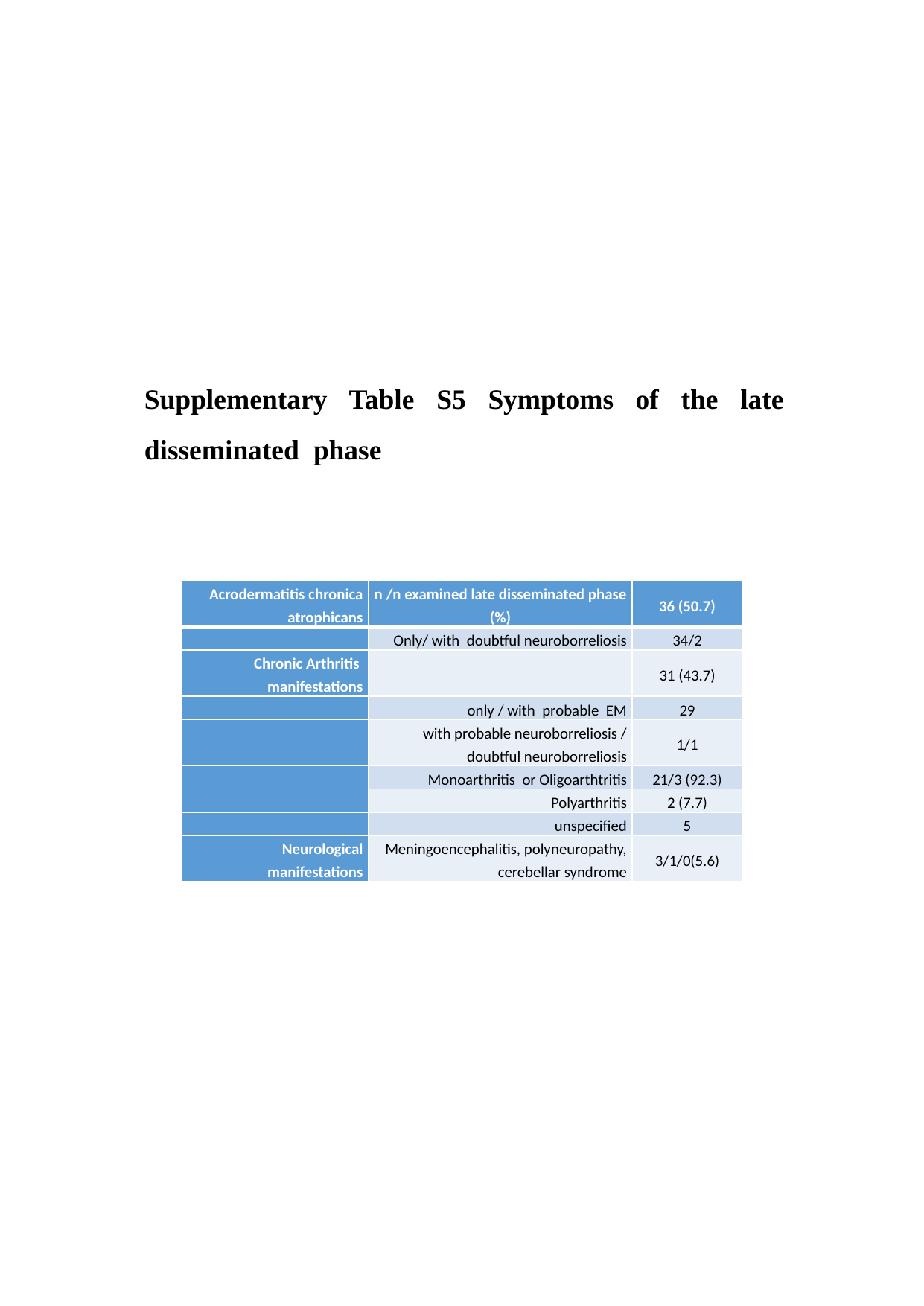

Supplementary Table S5 Symptoms of the late disseminated phase
| Acrodermatitis chronica atrophicans | n /n examined late disseminated phase (%) | 36 (50.7) |
| --- | --- | --- |
| | Only/ with doubtful neuroborreliosis | 34/2 |
| Chronic Arthritis manifestations | | 31 (43.7) |
| | only / with probable EM | 29 |
| | with probable neuroborreliosis / doubtful neuroborreliosis | 1/1 |
| | Monoarthritis or Oligoarthtritis | 21/3 (92.3) |
| | Polyarthritis | 2 (7.7) |
| | unspecified | 5 |
| Neurological manifestations | Meningoencephalitis, polyneuropathy, cerebellar syndrome | 3/1/0(5.6) |

## Slide 6
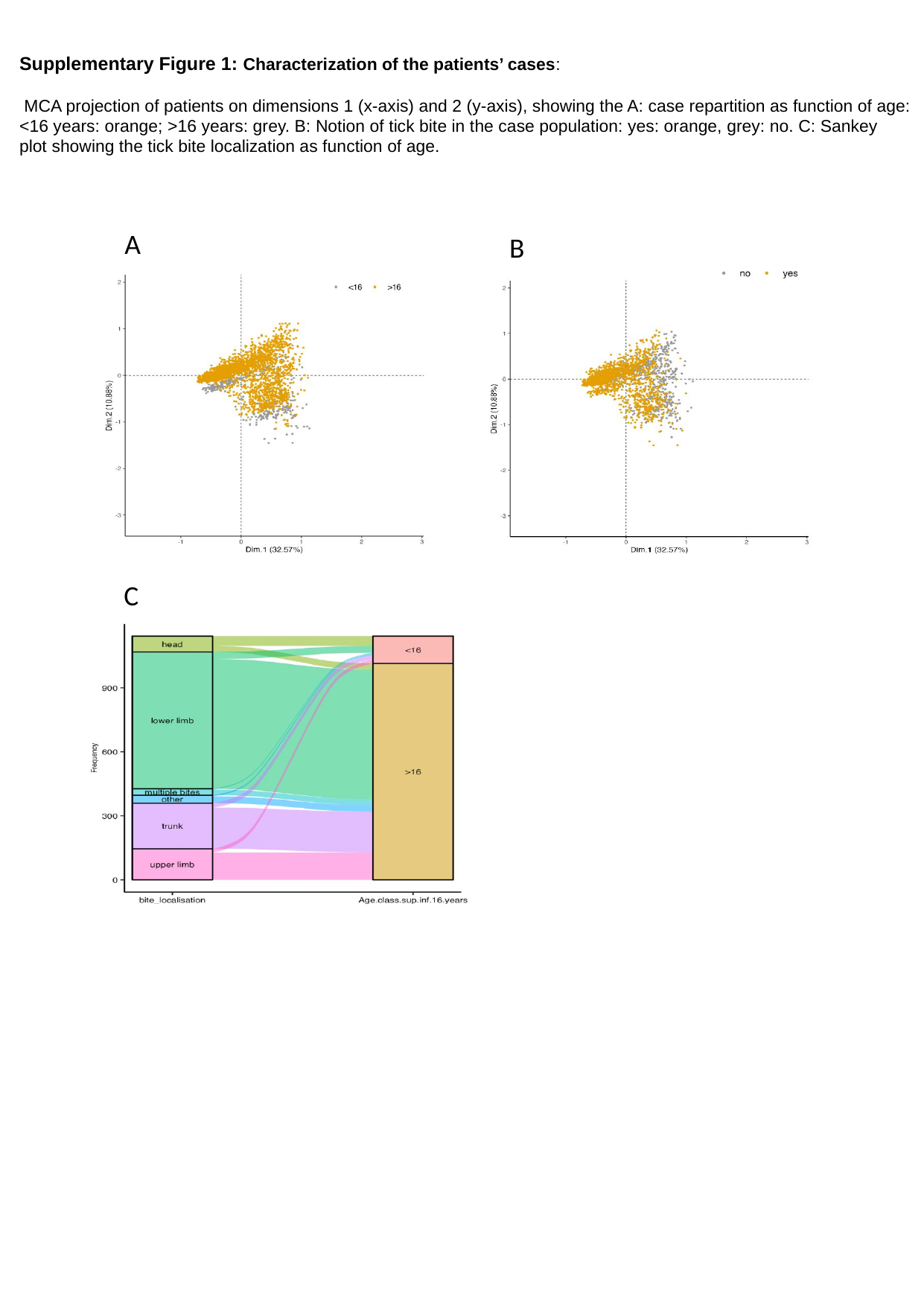

Supplementary Figure 1: Characterization of the patients’ cases:
 MCA projection of patients on dimensions 1 (x-axis) and 2 (y-axis), showing the A: case repartition as function of age:
<16 years: orange; >16 years: grey. B: Notion of tick bite in the case population: yes: orange, grey: no. C: Sankey
plot showing the tick bite localization as function of age.
A
B
C

## Slide 7
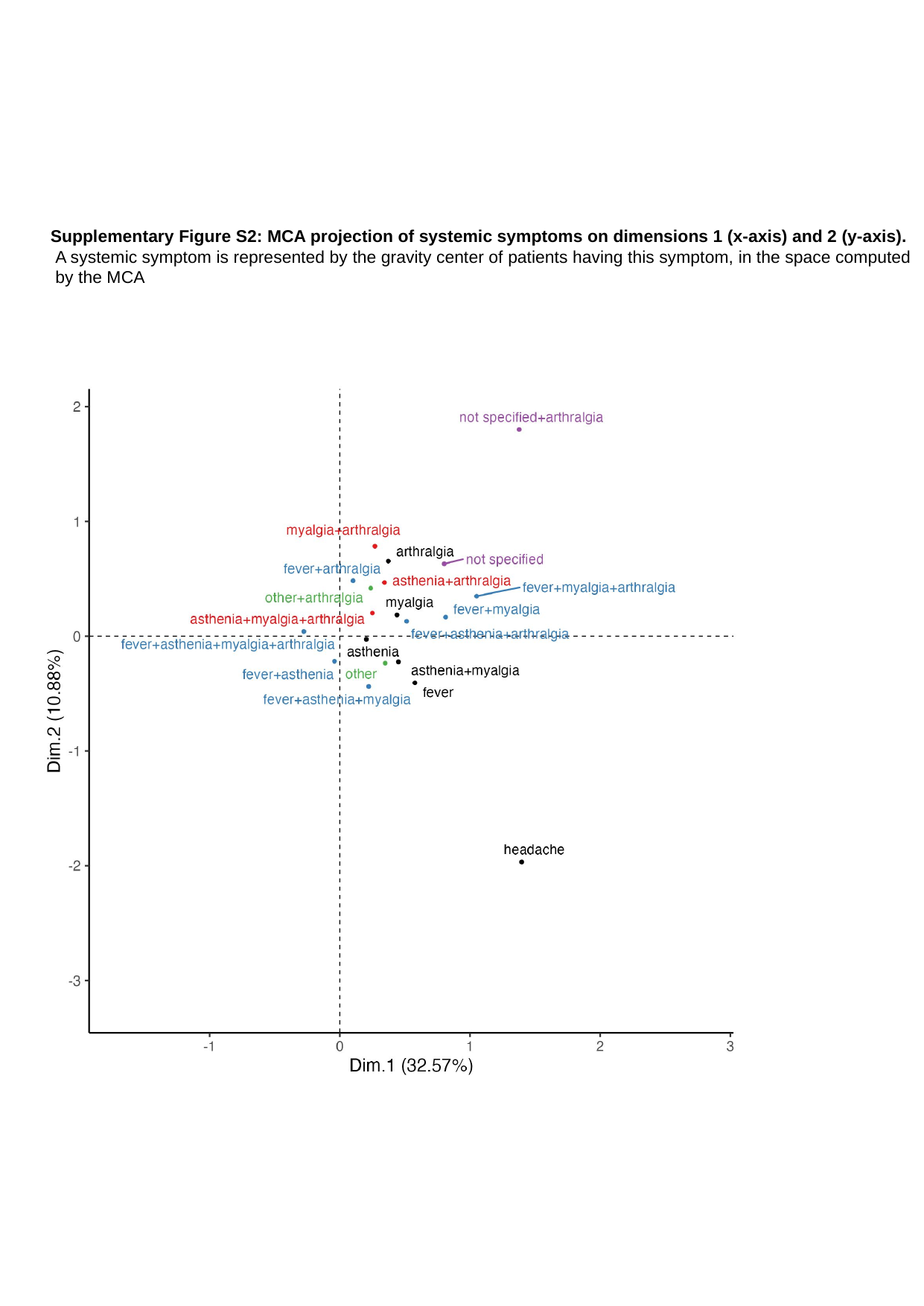

Supplementary Figure S2: MCA projection of systemic symptoms on dimensions 1 (x-axis) and 2 (y-axis).
 A systemic symptom is represented by the gravity center of patients having this symptom, in the space computed
 by the MCA

## Slide 8
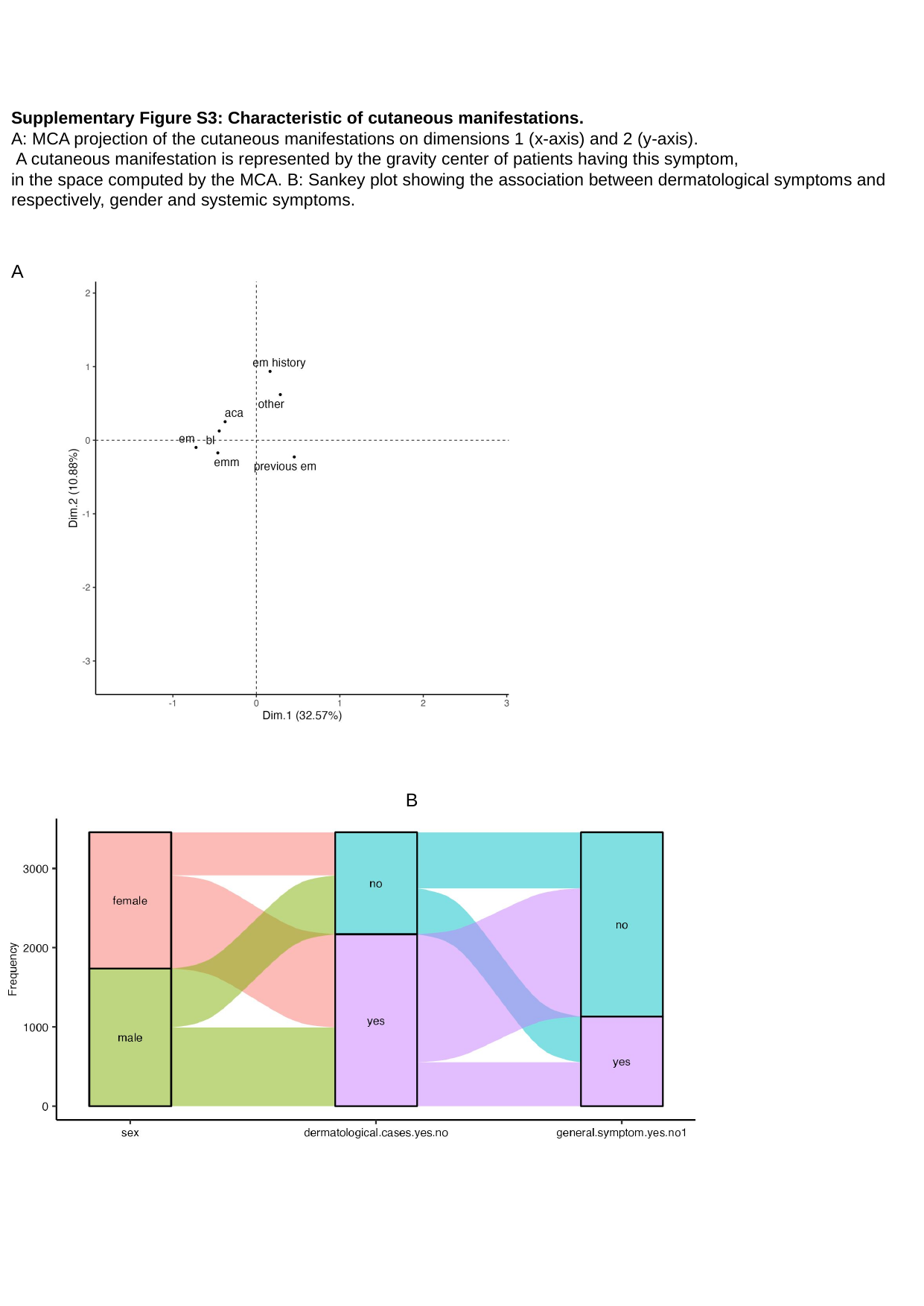

Supplementary Figure S3: Characteristic of cutaneous manifestations.
A: MCA projection of the cutaneous manifestations on dimensions 1 (x-axis) and 2 (y-axis).
 A cutaneous manifestation is represented by the gravity center of patients having this symptom,
in the space computed by the MCA. B: Sankey plot showing the association between dermatological symptoms and
respectively, gender and systemic symptoms.
A
B

## Slide 9
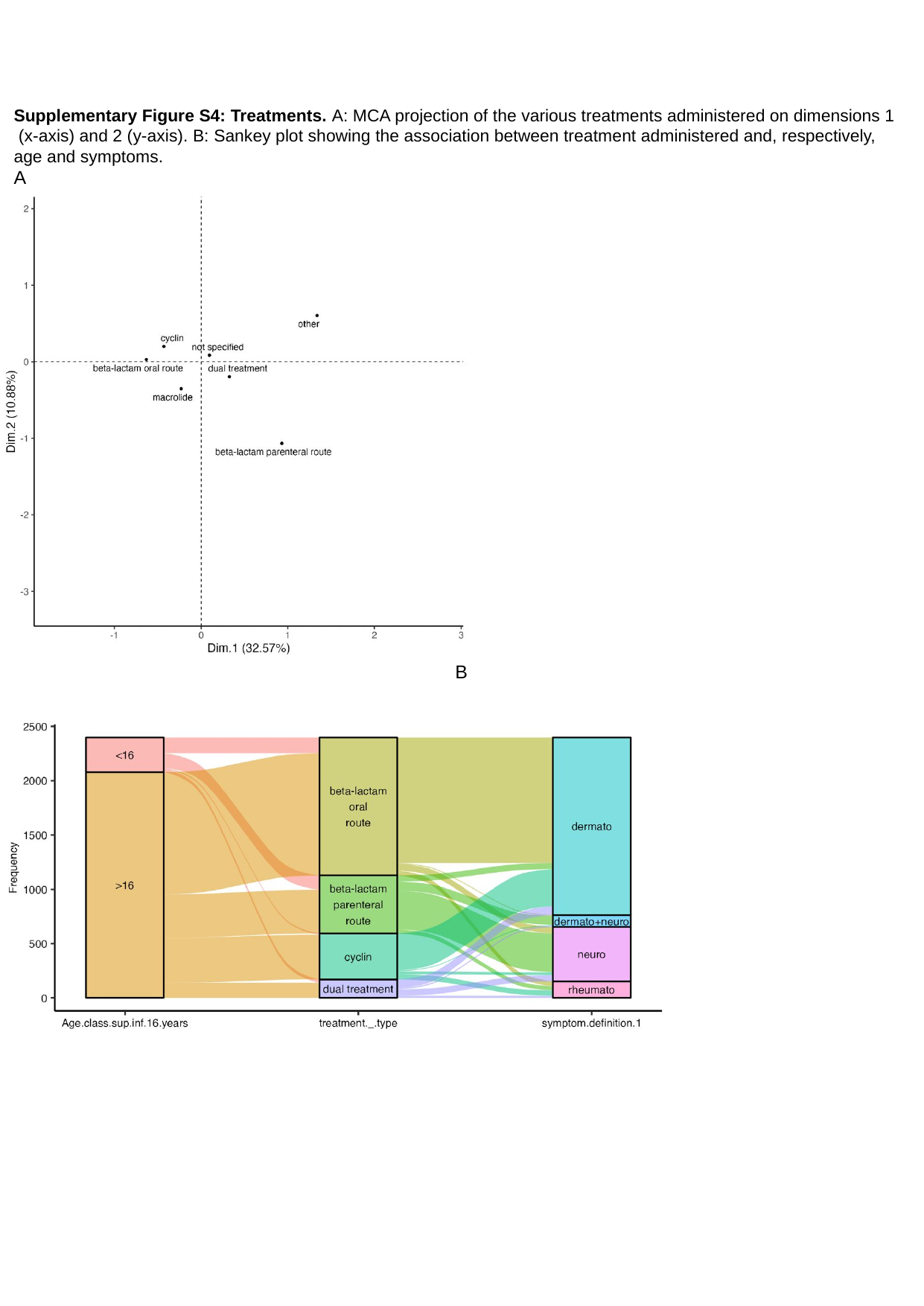

Supplementary Figure S4: Treatments. A: MCA projection of the various treatments administered on dimensions 1
 (x-axis) and 2 (y-axis). B: Sankey plot showing the association between treatment administered and, respectively,
age and symptoms.
A
B
